# Smart Nanomaterials in Cancer Theranostics: Challenges and Opportunities

**DOI:** 10.1021/acsomega.2c07840

**Published:** 2023-04-10

**Authors:** Brijendra Kumar Kashyap, Virendra Vikram Singh, Manoj Kumar Solanki, Anil Kumar, Janne Ruokolainen, Kavindra Kumar Kesari

**Affiliations:** † Department of Biotechnology Engineering, Institute of Engineering and Technology, Bundelkhand University, Jhansi 284128, Uttar Pradesh, India; ‡ Defence Research and Development Establishment, DRDO, Gwalior 474002, Madhya Pradesh, India; § Faculty of Natural Sciences, Plant Cytogenetics and Molecular Biology Group, Institute of Biology, Biotechnology and Environmental Protection, University of Silesia in Katowice, 40-007 Katowice, Poland; ∥ Department of Life Sciences, School of Natural Sciences, Central University of Jharkhand, Cheri-Manatu, Karmre, Kanke 835222, Ranchi, India; ⊥ Department of Applied Physics, School of Science, Aalto University, 02150 Espoo, Finland; # Faculty of Biological and Environmental Sciences, University of Helsinki, Vikkinkaari 1, 00100 Helsinki, Finland

## Abstract

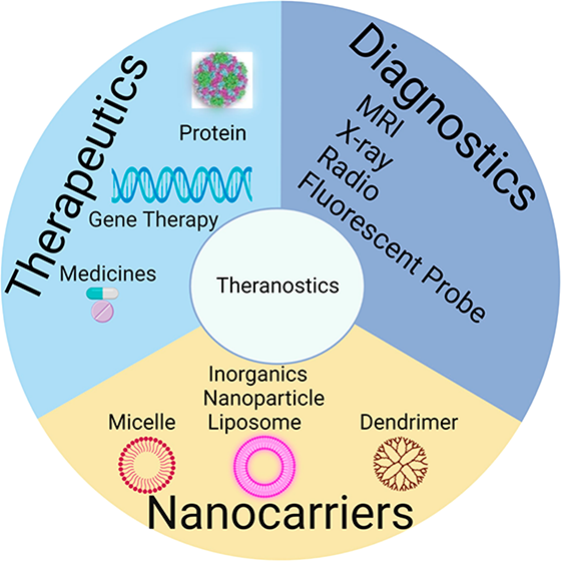

Cancer is ranked as the second leading cause of death globally. Traditional cancer therapies including chemotherapy are flawed, with off-target and on-target toxicities on the normal cells, requiring newer strategies to improve cell selective targeting. The application of nanomaterial has been extensively studied and explored as chemical biology tools in cancer theranostics. It shows greater applications toward stability, biocompatibility, and increased cell permeability, resulting in precise targeting, and mitigating the shortcomings of traditional cancer therapies. The nanoplatform offers an exciting opportunity to gain targeting strategies and multifunctionality. The advent of nanotechnology, in particular the development of smart nanomaterials, has transformed cancer diagnosis and treatment. The large surface area of nanoparticles is enough to encapsulate many molecules and the ability to functionalize with various biosubstrates such as DNA, RNA, aptamers, and antibodies, which helps in theranostic action. Comparatively, biologically derived nanomaterials perceive advantages over the nanomaterials produced by conventional methods in terms of economy, ease of production, and reduced toxicity. The present review summarizes various techniques in cancer theranostics and emphasizes the applications of smart nanomaterials (such as organic nanoparticles (NPs), inorganic NPs, and carbon-based NPs). We also critically discussed the advantages and challenges impeding their translation in cancer treatment and diagnostic applications. This review concludes that the use of smart nanomaterials could significantly improve cancer theranostics and will facilitate new dimensions for tumor detection and therapy.

## 1 Introduction

The alarming increase in new cancer cases worldwide concerns people’s lives and physical well-being. Despite advancements in science and technology and drug discovery methods, cancer is still the second leading cause of death worldwide after cardiovascular diseases.^1^ The global survey, including the individual medical data of 37.5 million cancer patients diagnosed between 2000 and 2014, shows that overall cancer survival rates are improving, including those for cancers with high malignancies.^2^ Cancer is responsible for about one in six deaths worldwide. Global Cancer Statistics 2020 estimates that 19.3 million new cancer cases will be responsible for approximately 10 million deaths.^3^ It turns out that low- and middle-income countries account for over 70% of cancer-related fatalities globally. Five main dietary and lifestyle disorders, including tobacco use, high body mass index, inadequate intake of fruits and vegetables, inactivity, and high alcohol consumption, are responsible for one-third of cancer-related fatalities.^4^ The complex, multifaceted origin makes cancer treatment more difficult. Additionally, many cancer treatments fail because of the development of multidrug resistant cells. Consequently, a single line of treatment cannot apply to all patients. Recurrence is frequently possible because cancer may become resistant to therapies that formerly cured it successfully.^5^

Chemotherapy, radiotherapy, and surgical intervention are the first-line cancer treatments,^6^ and they have continued to be the most effective methods for cancer treatment for the last several decades. Still, they have not been able to cure the disease entirely and have important limitations including low tumor selectivity, systemic toxicity, off-target toxicity, multidrug resistance, and serious adverse effects on human health.^7,8^ In particular, if a prompt diagnosis is not carried out, the probability of treatment failure or tumor recurrence and metastasis increases. Effective and personalized treatment plans incorporating cancer diagnostic and therapeutic approaches are required to achieve excellent clinical results. Afterward, monoclonal antibody based therapy has been explored by utilizing tumor-specific antigens (TSAs) and tumor-associated antigens (TAAs).^9^ Antibody–drug conjugates (ADCs) are biopharmaceutical drugs designed for targeted cancer therapy and sparing healthy cells. This approach has also paved the way from the bench side to the bedside in a majestic way.^10^ Despite the substantial progress of these techniques, the aforementioned techniques have their own advantages and disadvantages. To address this, researchers and scientists have worked on several methods and techniques, including organic chemistry, supramolecular chemistry, nanotechnology, oncology, and pharmacology.^11^ Nanotheranostics harnesses the capabilities of nanotechnology; due to their small size and leaky tumor vascularization, nanosystems can preferentially aggregate in tumor cells and exhibit enhanced therapeutic efficacy and diagnostic capability. Additionally, these nanoparticles (NPs) can be redirected and reoriented in a variety of ways while carrying the medicines on them.^12,13^ The emerging area of nanotechnology brings the concept of nanotherapeutics, and this technique has proved very promising in cancer treatment. Nanotechnology has been considered as the engineering of the molecule at the nanoscale level, which endows high drug loading capabilities due to high external surface area.^14^ Moreover, owing to the small size which is comparable with the biological system, nanomaterials actively interact with cellular components of the cell and show promising application in both *in vivo* and *in vitro* biomedical applications.

The fusion of biology with nanotechnology has been hailed as a revolutionary technological advance with numerous applications, including diagnostic devices, biosensing, drug delivery systems, and specialized therapeutic treatments.^15,16^ As a result of the recent advancements in the nanotechnology field, researchers are developing NP-based transport systems for the simultaneous delivery of diagnostic and therapeutic medications. The ability to codeliver a variety of therapeutic medicines and imaging agents is made possible by the small size of nanomaterials, which endows them with high surface areas and high drug-loading capacities.^17^ NPs can passively collect in tumors due to their well-known increased permeability and retention effects because dysfunctional arteries in tumor tissues cause aberrant molecular and fluid transport dynamics.^18^ Advance research focuses on discovering ground-breaking innovations in nanostructured medical devices with creative capabilities in diagnostic and preventative health.^19,20^

Drug resistance and tumor heterogeneity remain significant obstacles to effective therapy for cancer. Early and precise cancer detection is essential for the most potent therapeutic benefit after intervention.^21^ These critical aspects of the disease should be addressed by effective cancer treatment. A treatment that combines focused therapy based on precise diagnostic test results is known as theranostics, an emerging discipline of medicine.^22^ Hence, strategies combining simultaneous diagnosis and treatment are known as theranostics, enabling simultaneous target detection, drug distribution tracking, and therapeutic response evaluation to produce personalized medicine.^23^ As per Warner,^24^ theranostics is diagnosis along with therapy, which means it is an integrated approach that provides therapy, diagnosis, and monitoring through imaging.

The field of theranostic nanosystems is promising and extensive, and it warrants further research for the discovery of effective theranostic NPs, which enable a more personalized approach to nanomedicine. To this, NPs can be coated with hydrophilic materials such as folate, polyethylene glycol (PEG), hyaluronic acid, transferrin, aptamers, and antibodies that make NPs hydrophilic, which in turn increases the period of drugs, targeting efficiency by specific recognition, and enhances their penetration and accumulation in tumors.^25^ Numerous NPs also act as imaging agents due to the unique physicochemical properties of nanomaterials, along with improved transport. Hence, they need not be loaded with an additional imaging agent.^26,27^ For instance, the use of iron oxide in magnetic resonance imaging (MRI) allows diagnosis and therapy to be carried out concurrently rather than before or after therapy.^28^ The unique properties of noble metal Au and Ag nanoparticles show unique tunable optical properties because of their surface plasmon resonance (SPR) and have a strong penetrating ability, which makes them appropriate for theranostic applications.^29,30^ Thus, as theranostic agents, nanomaterials provide a unique advantage, including passive or active accumulation in tumor tissues and monitoring all activities within one formulation, ultimately reducing the patient’s inconvenience and potential adverse effects on the human body. The challenge associated with nanomaterials is their toxic properties, which should be addressed before their administration in clinical applications.

This review describes the documented literature on the recent progress of nanotechnology-based theranostic systems that have been extensively employed to support numerous clinical and preclinical cancer treatments. This provides an overview of nanomaterials used for cancer theranostics platforms along with a brief introduction to nanomaterial synthesis and its types, and attempts were also made to summarize the comprehensive novel nanotheranostic systems that have the potential for simultaneous cancer diagnosis and treatment in clinical translations followed by future perspectives and challenges. We conclude this review with a retrospective outlook of this important field and identify potential implications of this field’s paradigm in healthcare. With continued innovation and serious attention to the key challenges, it is expected that this important field will play a pivotal role in cancer diagnosis and treatment in clinical translations. We believe the summary of recent developments for the use of nanomaterials in cancer theranostics will provide a comprehensive understanding of the applications of NPs in biomedical research investigations and clinical uses.

## 2 Recent Progress and Perspectives of Smart Nanomaterials Based Theranostics

Theranostics is a unique concept that integrates therapy and diagnosis in a single system to achieve an accurate cancer diagnosis. It has been recognized as a potential breakthrough in resolving the problems with conventional oncotherapy.

**Figure 1:**
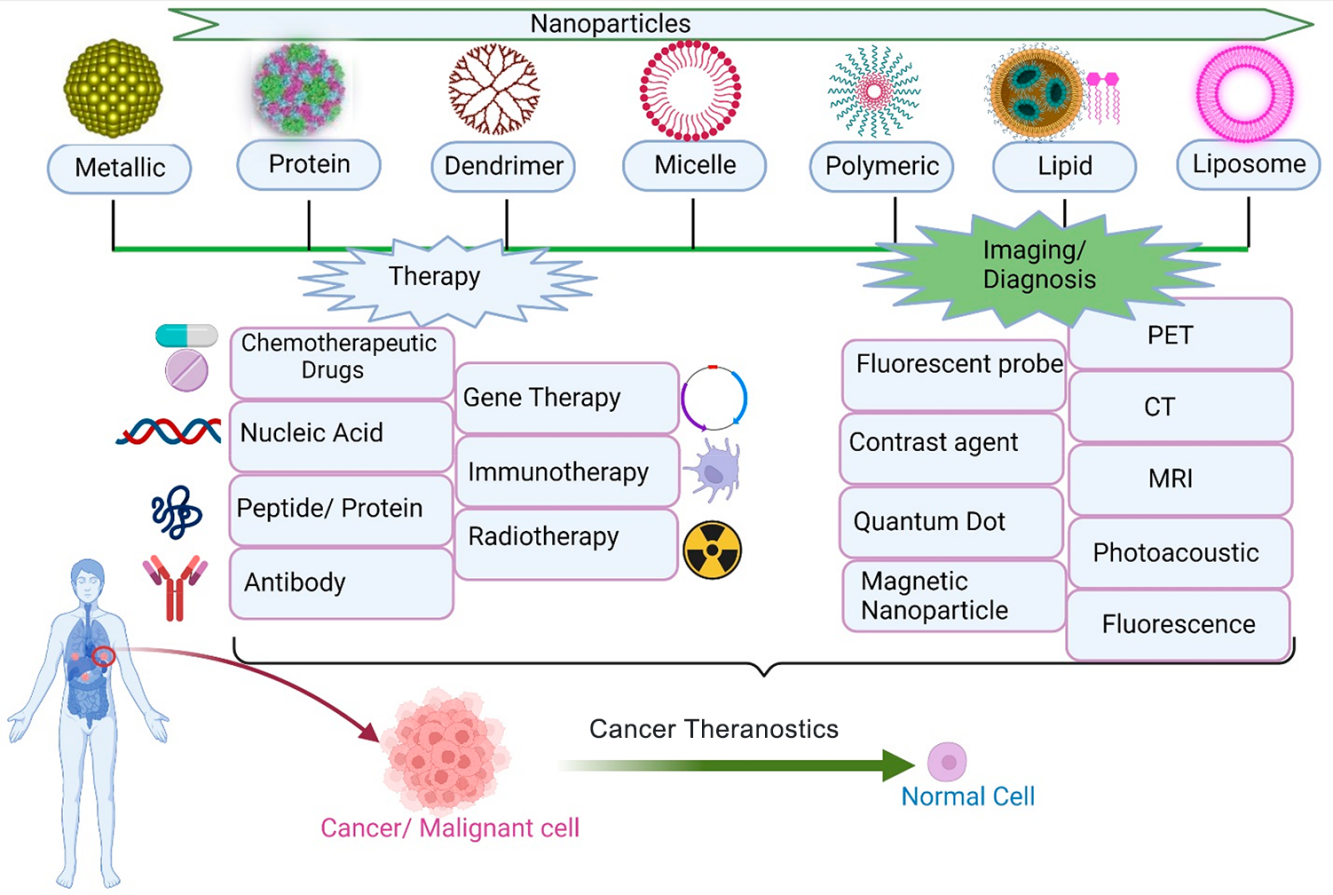
Nanotheranostic platform for simultaneous therapy and diagnosis. Comprehensive smart nanoplatforms from organic, inorganic, and carbon-based nanoparticles for cancer theranostics. PET, positron emission tomography; CT, computer tomography; MRI, magnetic resonance imaging.

Nanoparticles are ideal candidates as carriers for theranostic agents due to their exceptional physicochemical characteristics, such as nanoscale sizes, functional properties, active or passive tumor targeting, specific cellular uptake, and excellent optical properties that perfectly meet the needs of phototherapy and imaging at the same time. Metals and biological materials have significantly advanced cancer therapy and personalized medicine with the advancement of nanotechnology and medical technology. Theranostics has advanced significantly with the advancement of nanotechnology and is currently in the “bench to bedside” transition phase. In this review, we summarize recent progress on nanotechnology-based theranostics, i.e., nanotheranostics, that has significantly surpassed conventional therapies and has provided new therapeutic strategies, as well as “cocktail” theranostics (mixing various treatment modalities), as shown in Figure 1.

### 2.1 Applications of Nanomedicines and Targeted Nanotheranostics in Cancer Therapy

Chemotherapeutic medications suffer from several problems, including significant side effects and poor therapeutic efficacy.^31^ Nanomedicines help to improve the biodistribution and target accumulation of chemotherapy drugs, which allows them to better balance the effectiveness and toxicity of the treatment.^32^ Many nanomedicines have been investigated throughout the years, including liposomes, polymer–drug conjugates, and polymeric assemblies, to enhance tumor-targeted drug delivery.^33,34^ These nanomedicines use passive targeting, active targeting, and triggered release techniques. Recently, nanoscale biomaterial based technologies have resolved several complicated and challenging issues in science and technology.

Nevertheless, they also opened a new window in biomedical research focusing on personalized medicine. Nanoparticles are one of the greatest scientific breakthroughs because they have unique properties, including a high specific surface area and physicochemical features like optical, magnetic, electronic, catalytic, and antibacterial qualities.^35^ To combat the negative effects of cancer treatment, it is crucial to transport therapeutic medication molecules to the targeted tumor site. To enable site-specific cancer therapy, numerous efforts have been made over the past 20 years to develop drug delivery systems based on nanomaterials.^36^ Theranostics integrates therapy and diagnostics in one system which helps in developing an improved understanding of the treatment for its side effects and benefits and captures an image while dispensing therapeutic medications at a precise dosage.^37^

An overview of recent advancements in targeted nanobased cancer treatments is provided in this review article, along with a variety of diagnostic probes and therapeutic medications. Moreover, syntheses and applications of inorganic nanoparticles, carbon nanoparticles, micelles, protein conjugates, linear and branching polymers, and dendrimers in theranostics are also covered. The use of theranostics in imaging methods like computed tomography, magnetic resonance imaging, single-photon emission computed tomography, and fluorescence/optical imaging for *in vivo* imaging was also highlighted. About a dozen nanomedicines based on polymeric micelles are currently undergoing clinical trials for various cancers. The latter is desirable for delivering chemotherapeutic drugs with low water solubility.^38^ Integrating therapy with noninvasive imaging is extremely valuable for understanding the *in vivo* fate, pharmacokinetics, target site accumulation, and therapeutic efficacy of nanomedicines.^39^ By selecting patients in advance who are most likely to respond to nanotherapy, this information can be utilized to evaluate the suitability of nanotherapeutic treatments based on nanomedicine.^40^ The fundamental ideas of nanoparticle-based tumor targeting and clinical and preclinical cancer treatments from chemotherapy, to radiotherapy and to photodynamic therapy, and from photothermal therapy to gene therapy, are outlined in this paper, along with the advantages of utilizing imaging to preselect patients and tailor nanomedicine therapies.

Being heterogeneous by nature, cancer is a complicated disease.^41^ Despite notable advancements in the development of new drugs and conventional treatment modalities, still the success rate of cancer survival remains undermined.^42^ Drug resistance and cancer heterogeneity are the main obstacles that reduce a drug’s efficacy. A patient-centric or individualized approach is required for a poor medication response for a better therapeutic result.^43^ Nanotheranostics has become a promising way to handle such complex problems which amalgamates therapy and diagnosis.

These advanced nanocarriers are capable for drug targeting while enabling diagnostic attributes like imaging.^44^ Recent studies indicate that the cancer nanotheranostics platform provides improved pharmacology of the drugs leading to low toxicity compared to conventional approaches.^45^ Passive or active, the targeting of drugs by nanoparticle-based drug delivery systems (NDDSs) achieves a better distribution of the drug to the tumor sites, which shows a great advantage in cancer therapy.^46^ A nanoparticle-based theranostic agent is rendered more attractive for personalized therapy because it integrates the three perspectives—diagnosis, drug delivery, and treatment response monitoring—in a single platform. Nanotheranostics, in combination with the targeted drug, could provide great potential for an individualized therapeutic paradigm for cancer. In addition, NDDSs could also combine distinct therapeutic modalities into a single feasible platform to offer synergistic effects and also to reverse the development of drug resistance.^47^ Lipid-based nanoparticles, polymers, dendrimer-based nanoparticles, metal nanoparticles (noble metals such as gold and silver), semiconductor nanoparticles, carbon nanotubes, metal oxide nanoparticles, metal–organic frameworks (MOFs), and up-converting nanoparticles (UCNPs) are NDDSs which are available for cancer therapy.^48–52^ Cancer nanotheranostics aims to utilize nanotechnology to combine cancer therapy and imaging. Engineering nanomaterials can greatly enhance the effectiveness and specificity of therapy for cancers to interact with cancer cells at the molecular level.^12^ The greatest therapeutic challenge for targeted therapy is posed by metastasis, drug-resistant cancers, and cancer stem cells.^53^ Nanoparticles, adult stem cells, or T-cells in immunotherapy can appropriately be developed as drug delivery systems to achieve a targeted therapy.^54^

## 3 Synthesis of Nanomaterials

**Figure 2:**
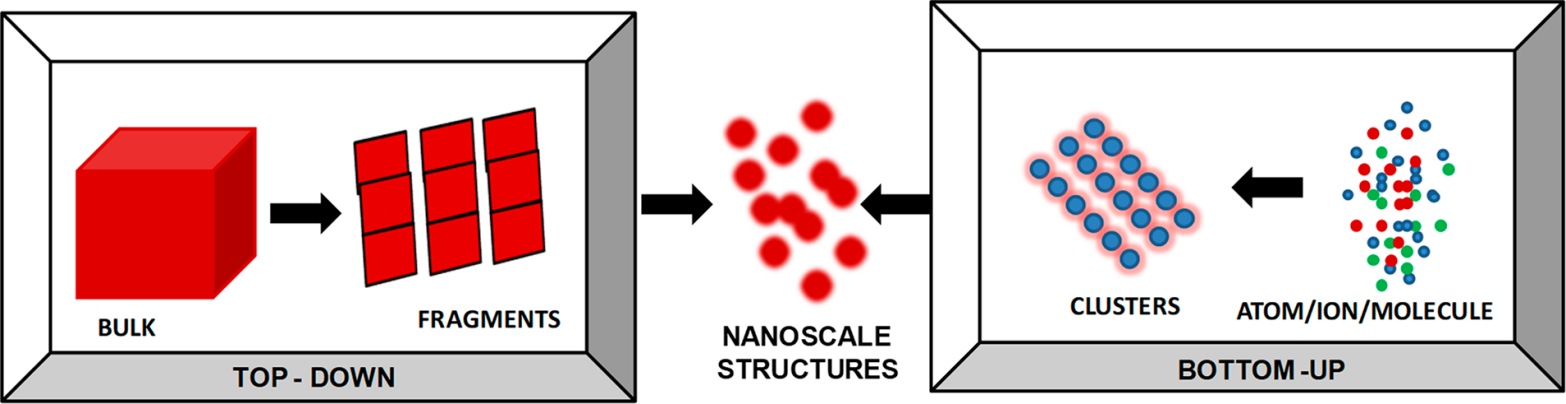
Synthesis of nanomaterials: top-down and bottom-up approaches.

The various techniques used to synthesize the nanomaterials can be broadly categorized as bottom-up and top-down, as shown in Figure 2.

### 3.1 Bottom-Up Approach

This method is called a constructive approach for synthesizing nanomaterials. Common bottom-up approaches are sol–gel, spinning, chemical vapor deposition (CVD), pyrolysis, and biosynthesis.

#### 3.1.1 Chemical Vapor Deposition Technique (CVD)

In the chemical vapor deposition method, a thin film is deposited onto a substrate via the chemical reaction of the gaseous substrate. This reaction is generally carried out at ambient temperature. A thin film is formed by the chemical reaction which occurs when a heated substrate comes into contact with the precursor gas. This method can obtain a uniform, pure, rigid, and robust film with good reproducibility. One of the major disadvantages of this technique is the requirement of special equipment and gaseous byproducts, which are sometimes highly toxic.^12^

#### 3.1.2 Solvothermal and Hydrothermal Methods

Solvothermal and hydrothermal methods are popular conventional approaches for synthesizing nanomaterials such as nanowires, nanorods, nanosheets, and nanospheres. In the solvothermal process, the reaction is carried out in a closed sealed vessel whose pressure should be greater than the solvent’s boiling point. In the hydrothermal process, instead of solvent, water is used. The microwave-assisted hydrothermal approach combines the merit of microwave and hydrothermal methods. In this approach, temperature affects the morphology of the nanomaterials.

#### 3.1.3 Sol–Gel Method

The sol–gel method is one of the most widely used wet chemical approaches for synthesizing nanomaterials owing to its simplicity, economical viability, and environmental benignity. As the name suggests, in this method, there is the dissolution of precursor in a solvent system which is transformed into the sol, and the sol is finally converted into gel through the process of condensation, hydrolysis, gel aging, etc. Metal oxides and chlorides are the conventional precursors for this method. During the condensation process, hydroxo (M–OH–M) or oxo (M–O–M) bridges synthesize metal–hydroxo or metal–oxo clusters in solution. The material’s structure, characteristics, and porosity change due to ongoing polycondensation. Aging results in a reduction in porosity and an increase in the spacing between colloidal particles and formation of a gel. After the aging process, the gel is dried, which removes water and organic solvents from it, followed by calcination to get nanoparticles.

#### 3.1.4 Pyrolysis

In industry, pyrolysis is a well-used technology for the large-scale synthesis of nanomaterials. Herein, the precursor is fed into the furnace at high pressure through a small orifice and burned in the flame at a high temperature involving the thermal decomposition of materials in an inert atmosphere. This method is simple, efficient, and economically viable with a high yield.

#### 3.1.5 Biosynthesis

This is the greener approach for synthesizing nontoxic and biodegradable nanoparticles. Instead of using conventional chemicals for bioreduction and capping, the biosynthesis method produces NPs using bacteria, plant extracts, fungi, and other microorganisms together with the precursors. Because of their distinctive and improved features, biosynthesized NPs are used in various applications, including drug carriers for targeted delivery, cancer treatment, gene therapy, DNA analysis, antibacterial agents, biosensors, separation science, and magnetic resonance imaging (MRI).

#### 3.1.6 Reverse Micelle Method

The reverse micelle method is an intriguing wet chemical method that can be used to synthesize nanomaterials with the desired shape and size. In this process, reverse micelles are formed from at least three components; two are immiscible and the third is a surfactant with amphiphilic properties. Aqueous systems with nanometer dimensions are used to carry out specific reactions in order to develop materials with controlled size and shape. For controlling the size of the nanomaterial, the reverse micelles’ size plays a crucial role. In this method, the reverse micelle acts as a nanoreactor, leading to an enhanced reaction rate and uniform distribution of NPs. The advantage of this approach over other approaches lies in improved control of particle size, shapes, uniformity, and dispersibility.

### 3.2 Top-Down Method

In contrast to bottom-up approaches, top-down approaches reduce bulk materials to nanoscale particles. As a result, this method is also known as a destructive method for nanoparticle synthesis. Some of the common synthetic methods for this approach include lithography, mechanical milling, laser ablation, sputtering, and thermal decomposition.

#### 3.2.1 Lithography

A focused electron beam is used to synthesize nanomaterials. This approach is broadly classified into two main categories: masked lithography and maskless lithography. Generally, lithography is used in microfabrication to pattern part of a thin film using the bulk substrate.

#### 3.2.2 Mechanical Milling

Among the various top-down approaches, mechanical milling is a physical method for synthesizing various nanoparticles. Milling aims to reduce the particle size and blend the particle in an inert atmosphere. Plastic deformation determines the particle shape and fracture and reduces particle size, and cold welding, which increases particle size, is the influencing factor in mechanical milling. This method is simple, is low cost, and can produce various nanoparticle sizes.

#### 3.2.3 Laser Ablation

This is a common method for fabricating a wide range of nanomaterials, including semiconductors, metal NPs, nanowires, composites, ceramic carbon nanotubes, etc., from various solvents. In this approach, a laser beam is irradiated on a metal solution. Due to the high laser energy, the precursor vaporizes, followed by nucleation and growth of a plasma plume, which leads to the synthesis of nanoparticles. This technique is considered a green process as it obviates the need to stabilize and reduce agents for the NP synthesis.

#### 3.2.4 Sputtering

In this process, high-energy particles bombard the solid surface, which leads to the production of NPs. It is a very effective technique for producing a thin film. Sputtering is usually a deposition of a thin layer of materials onto a particular surface by ejecting atoms from that material and condensing ejected atoms onto the surface when a high vacuum environment is applied.^55^ Sputtering can be carried out in a number of ways, including radio frequency diodes, magnetrons, and DC diodes. Sputtering is often carried out in an evacuated chamber that is then filled with sputtering gas. Free electrons collide with the gas due to a high voltage supplied to the cathode target, creating gas ions. Atoms are ejected off the cathode target’s surface due to the positively charged ions’ rapid acceleration in the electric field as they approach the target.^56^ The thickness of the layer, temperature, duration of annealing, substrate type, etc., determine the shape and size of the synthesized film over the substrate.^57^

## 4 Classification of Nanomaterials

Nanoparticles can be classified according to their physical and chemical properties. Generally, NPs can be classified into organic, inorganic, and carbon-origin nanoparticles.^58^

### 4.1 Organic Nanoparticles

**Figure 3:**
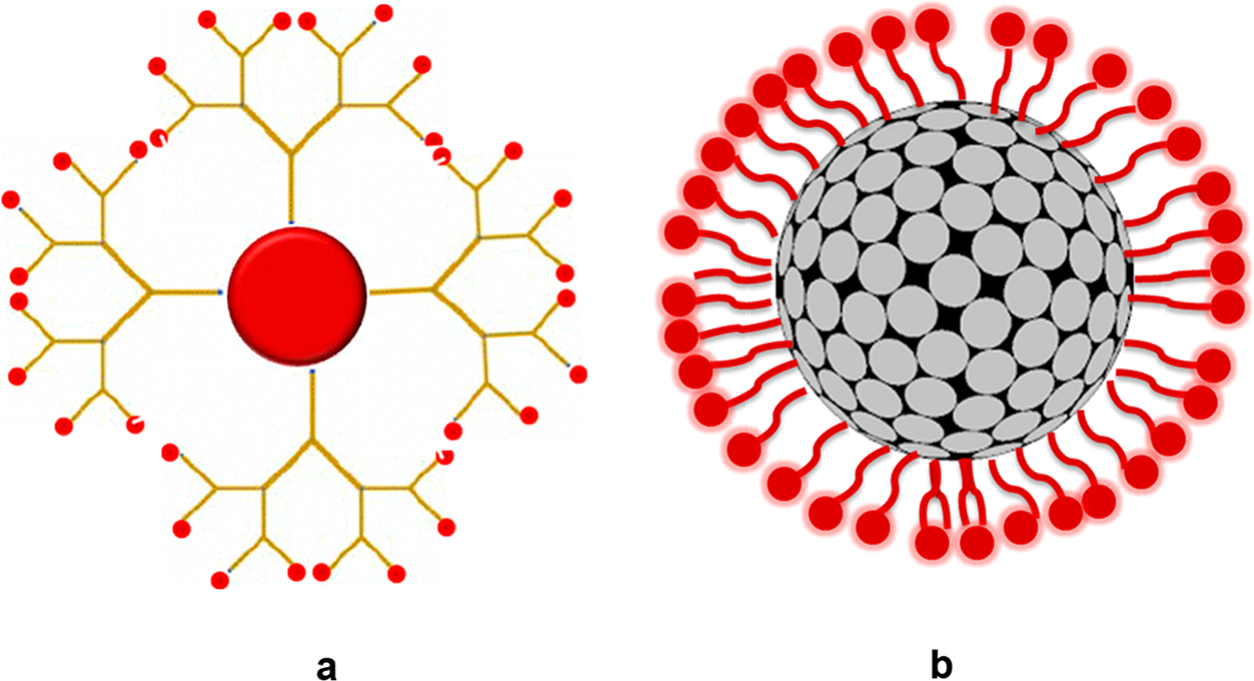
Organic nanoparticles: (a) dendrimer and (b) liposome.

Organic nanoparticles are synthesized utilizing natural or synthetic organic molecule templates, as shown in Figure 3. There are many different types of organic NPs in nature, including protein aggregates, lipid bodies, milk emulsions, and more complex structures like viruses, to mention a few.^59^ Common names for organic nanoparticles or polymers include dendrimers, micelles, chitosan, silk fibroin, liposomes, and ferritin. These nanoparticles are biodegradable and nontoxic, and some are like micelles and liposomes, which have hollow centers, so they are also called “nanocapsules”. The high stability of organic NPs in biological fluids and during storage makes them an ideal choice for stimuli-responsive materials triggered by electromagnetic radiation like heat and light.^59^ Biopolymer nanoparticles offer several advantages, including the ease of their preparation, higher colloidal stability, improved dispersibility, and surface reactivity, making them a perfect candidate for drug administration.^60^

### 4.2 Inorganic Nanoparticles

Inorganic nanoparticles are biocompatible, hydrophilic toxic, and highly stable. Some common examples of inorganic nanoparticles include quantum dots and metal and metal oxide nanoparticles. These inorganic NPs are highly efficient for diagnosis and imaging due to their unique electrical, chemical, and magnetic properties.^61−63^

#### 4.2.1 Metal-Based NPs

Metal nanoparticles are currently being used for biological applications. This covers a broad range of substances, including elemental metals such as cadmium (Cd), cobalt (Co), aluminum (Al), iron (Fe), copper (Cu), gold (Au), silver (Ag), lead (Pb), and zinc (Zn), as well as metal oxides and metal salts.^63,64^ Silver nanoparticles (AgNPs) and gold nanoparticles (AuNPs) show optical plasmonic behavior that leads to enhancement in the electromagnetic field due to collective oscillation of the electrons and accordingly enhances the radiative properties for cancer theranostics applications.^65,66^ Moreover, iron nanoparticles are exclusively used for magnetic resonance imaging (MRI) applications.^67^

#### 4.2.2 Metal Oxide NPs

Metal oxide NPs are well-known for their anticancer activity and enhanced radiosensitization ability. In order to modify the properties of their respective metal-based nanoparticles, metal oxide nanoparticles are synthesized. For example, in the presence of oxygen at room temperature, iron (Fe) nanoparticles instantly oxidize to iron oxide (Fe_2_O_3_), increasing their reactivity compared to iron NPs. It has also been proven that ZnO NPs are most effective in targeting certain cancer cells. The genotoxic ZnO NPs offer a promising platform for designing more potent anticancer agents for therapeutic use. The surface modification of metal oxides can be performed with carboxylate, oleic acid, and polyethylene glycol to deliver various therapeutic agents. Surface modifications of these NPs make them more biocompatible, reactive, and biodegradable by improving properties such as cytotoxicity, hydrophilicity, large pore volume, high surface area, and pore size. These modifications lead to an enhancement in therapeutic efficacy. Metal oxides such as aluminum oxide (Al_2_O_3_), iron oxide (Fe_2_O_3_), cerium oxide (CeO_2_), and zinc oxide (ZnO) are attracting particular interest due to their enormous potential in anticancer therapy.^68^ Particularly CeO_2_, by virtue of its redox-modulatory enzyme-like activities, is envisaged as a promising candidate in nanomedicine.^68−70^ The ability of cerium nanoparticles to mimic various redox activities, allowing them to scavenge reactive oxygen species, such as catalase, superoxide dismutase, peroxidase, phosphotriesterase, phosphatase, and oxidase, make it a potential candidate for cancer theranostics.

### 4.3 Carbon-Based NPs

**Figure 4:**
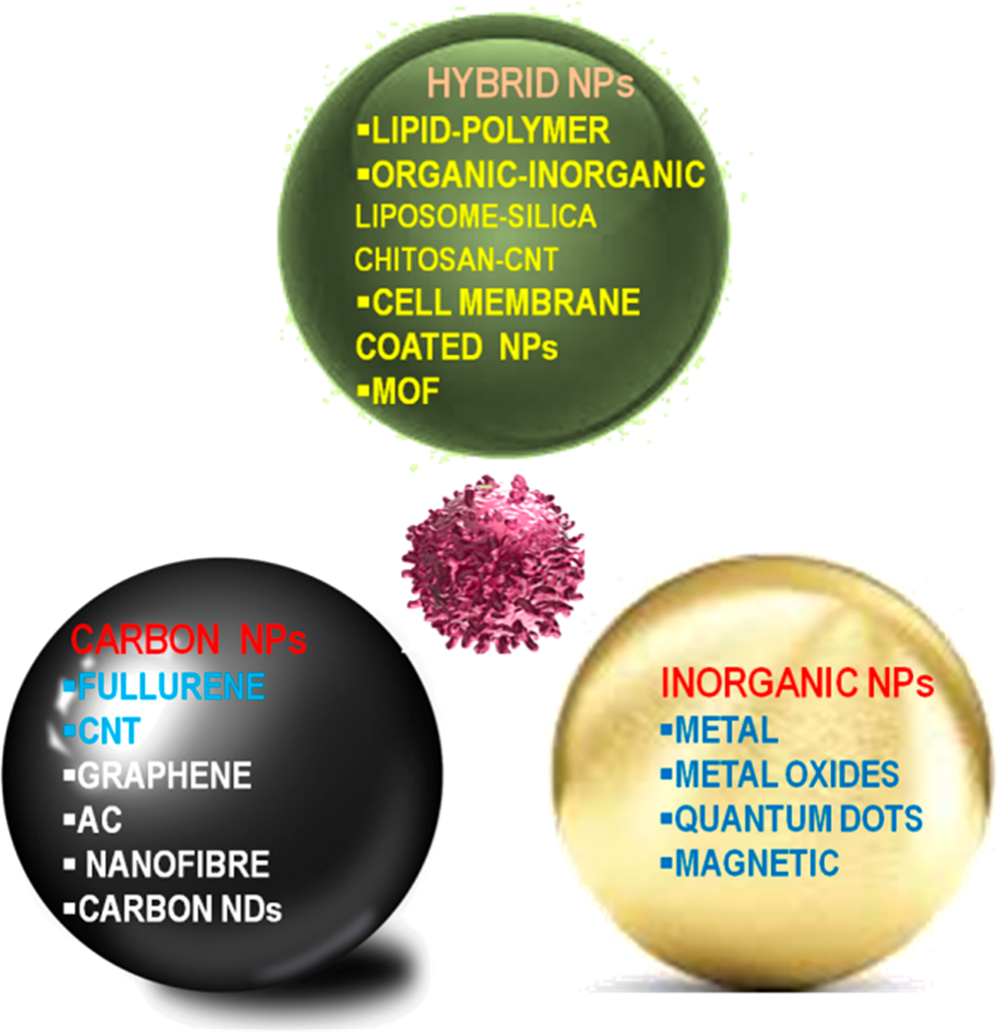
Different types of nanoparticles for cancer theranostics.

Carbon-based nanomaterials are extensively used in biomedical applications, particularly for cancer theranostics, thanks to their unique physicochemical properties and inert nature, which enhances their ability to deliver drugs to the cell without any adverse effects. The feasibility of surface functionalization with organic and biological molecules makes them some of the most promising materials capable of targeted drug delivery. Carbon nanomaterials show strong absorption in the near-infrared region (NIR), which is helpful for photothermal tumor ablation. Furthermore, these materials are capable of releasing heat in a radio frequency field, which can be utilized to kill cancer cells by heating them. As a result, they can be applied to photodynamic and photothermal therapies. Carbon-based nanomaterials can be classified into activated carbon, fullerenes, graphene, carbon nanotubes (CNTs), carbon nanofibers, carbon black, etc. as shown in Figure 4.

#### 4.3.1 Activated Carbon

Activated carbon (AC) is an amorphous form of carbon and is produced from a range of carbonaceous sources, such as bamboo, wood, coconut shells, and coal, by the process of carbonization and activation. AC is economically viable and biofriendly, making it an ideal candidate as a carrier for tumor therapeutic agents.

#### 4.3.2 Graphene

Graphene is a carbon allotrope and comes under two-dimensional materials. Graphene possesses extraordinary physicochemical properties, including ultrahigh carrier mobility, excellent electrical conductivity, superior thermal conductivity, large specific surface area, high optical transmittance, and good biocompatibility. Due to the above properties, graphene has been widely used as an excellent drug delivery system, in fluorescence imaging, and in cancer theranostics.

#### 4.3.3 Carbon Nanotubes

Carbon nanotubes (CNTs) are novel-type synthetic nanomaterials a few nanometers in diameter with distinct hollow and cylindrical structures. CNTs are formed by rolling graphene sheets. CNTs possess extraordinary chemical, electronic, mechanical, and optical properties. The functionalization of CNTs with other biological systems makes them ideal for biocompatible drug delivery strategies for targeting and eliminating specific tumor cells.

#### 4.3.4 Fullerene

A fullerene is also an allotrope of carbon. In a fullerene, carbon atoms are held together by sp^2^ hybridization and belong to the carbon nanomaterials family. The distinct cagelike structure and electron-deficient nature impart fascinating properties, making fullerenes a promising focus of various research areas, including cancer theranostics.

#### 4.3.5 Carbon Nanodots

Carbon nanodots (CNDs) are zero-dimensional nanoparticles having a size of less than 10 nm. CNDs are fluorescent materials and exhibit unique characteristics such as high water solubility, chemical inertness, low toxicity, ease of functionalization, and good biocompatibility. CNDs are fascinating carbon-based materials and have received much attention in bioimaging, optical sensing, anticancer, photocatalysis, lasers, drug delivery, and optoelectronics.

## 5 Recent Trends of Nanomaterials in Cancer Theranostics

Due to their low cost, simple synthesis, and minimal toxicity, bioinspired nanoparticles surpass traditionally synthesized nanoparticles by mimicking nature. This review article provides a comprehensive overview of cancer theranostics using a variety of bioinspired materials, which include lipid nanoparticles, protein-based nanoparticles, liposomes, inorganic nanoparticles, viral nanoparticles chitosan, and silk fibroin.^71−75^ Because of their dimensions in the range 1–100 nm, nanocarriers possess greater and more effective interactions with cancerous cells and are greatly explored for biomedical applications.^76^

**Figure 5:**
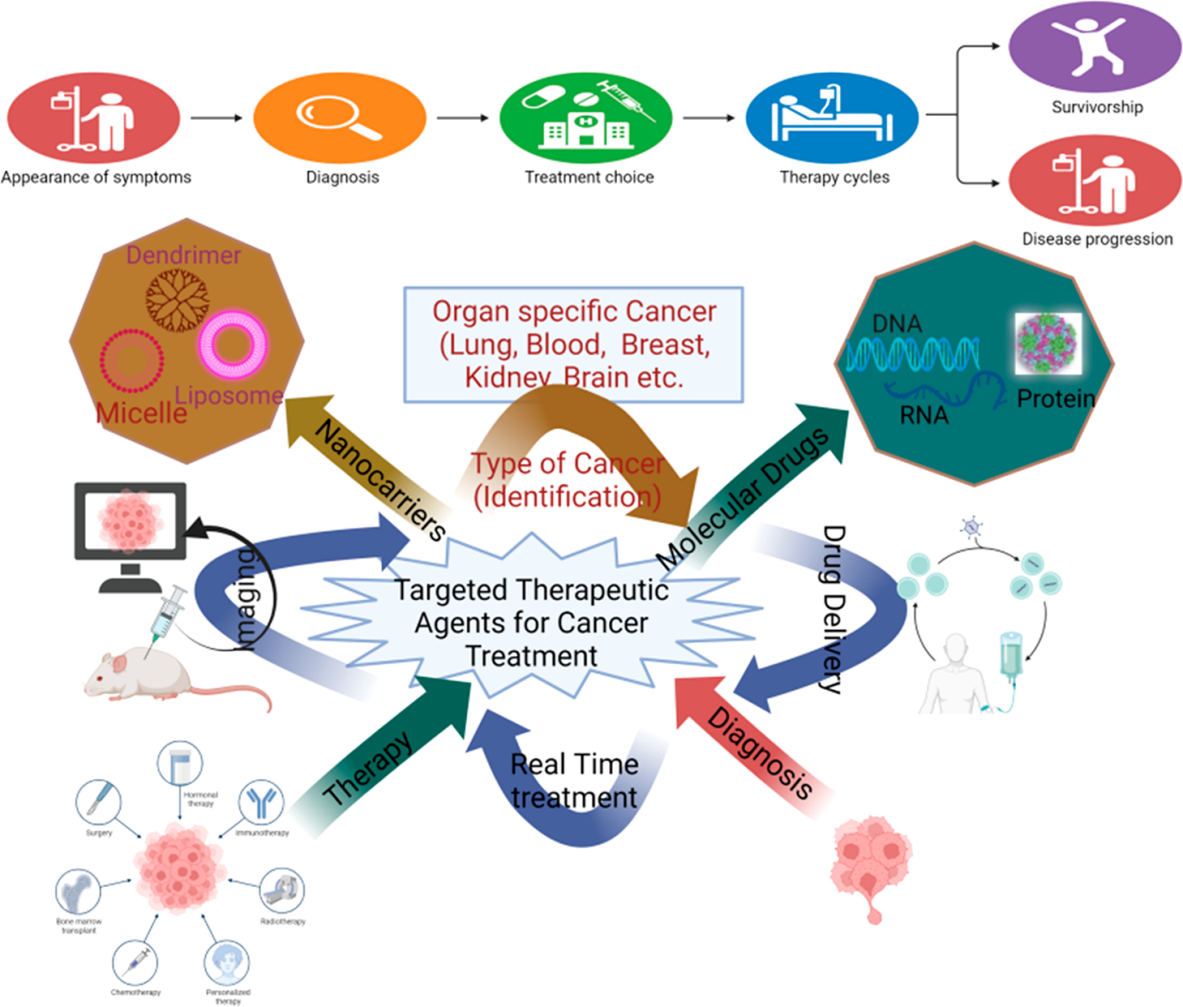
Recent trends in cancer theranostics: identification, drug delivery, real-time treatment, and imaging.

Due to their distinct physicochemical characteristics, bionanoparticles have received a lot of attention recently. Viral NPs, protein NPs, apoferritin, aptamers, solid-lipid NPs, etc.^21,77−80^ appear to be the most promising members of the cauldron. Recent trends in cancer theranostics are displayed in Figure 5.

However, it has been shown that biosynthesized multifunctional nanoparticles encapsulating therapeutic and imaging agents possess theranostic activity.^81^ The following approaches can synthesize a bioinspired theranostic agent: (i) screening of plant extracts; (ii) standardizing a variety of physicochemical parameters for biosynthesis; (iii) inclusion of therapeutic and imaging agents; (iv) characterization of nanocarriers to determine their properties.^82,83^

The hybridization of gold NPs with quantum dots (QDs) can produce multifunctional nanohybrids with superior imaging and anticancer properties. Chen et al. created ZnO QDs conjugated to gold nanoparticles containing the anticancer drug camptothecin.^84^ AuNPs convert absorbed light energy into localized heat responsible for tumor cells’ destruction via photothermal therapy. Nanocarriers, with and without drug loading, exhibited similar cytotoxicity toward HeLa cells. On the contrary, hybridization of the paramagnetic Gd ion and CuInS/ZnS QDs could enable dual fluorescence/magnetic resonance mediated imaging.^85^

Yang et al. synthesized Gd-doped ZnS QDs without Cd in lipid vesicles with enhanced fluorescence and improved colloidal stability. Similarly, to avoid fluorescence quenching of QDs, superparamagnetic Fe_3_O_4_ was separated from fluorescent graphene–CdTe QDs by a SiO_2_ shell.^86^ 5-Fluorouracil could be loaded into this bifunctional cytocompatible model, which would be effective against hepatoma cells. Overall, the hybridization of QDs with different types of inorganic NPs enables their use in multimodal imaging, such as fluorescence, magnetic, and ultrasound imaging. By combining magnetic hyperthermia, photothermal therapy, and photodynamic therapy, synergistic cancer therapy could also be achieved.^33^

### 5.1 Organic Nanoparticles in Cancer Theranostics

In the past decade, there have been significant increases in the application of nanotechnology to detect and treat numerous diseases, including cancer.^87^ Nanotechnology helps to improve the biodistribution and target accumulation of chemotherapy drugs, allowing them to balance better the efficacy and toxicity of the treatment.^88^ In response to the various limitations in conventional therapeutic strategies and to improve tumor-targeted drug delivery, various nanomedicines including liposomal nanoparticles, nonmetallic nanoparticles, viral nanoparticles, protein nanoparticles, and lipid nanoparticles have been studied.^89,90^ It is important to note that nanoparticles offer small size and enhanced drug loading capacity, ease of functionalization, simple penetration capabilities, and improved retention inside the targeted tissue which have significantly improved the diagnostics and therapeutics of various cancers. These nanomedicines use passive targeting, active targeting, and triggered release techniques. In the recent era, nanotechnology has resolved several complicated issues in the fields of science and technology.^91,92^ It may also open a new window in the biomedical realm focusing on personalized medicine. To combat the negative effects of cancer treatment, it is crucial to transport therapeutic medication molecules to the targeted tumor site. Numerous efforts have been made over the past 20 years to enable site-specific cancer therapy to develop drug delivery systems based on nanomaterials.^93^ Theranostics is a recently developed nanotechnology that describes a method of combining medicinal, diagnostic, and imaging methods into a single unit. Doxil is the first nanomedicine approved by the U.S. Food and Drug Administration (FDA).^94^ About a dozen nanomedicines based on polymeric micelles are currently undergoing clinical trials for various cancers. The latter is particularly attractive for delivering chemotherapeutic drugs with low water solubility.^95^ Integrating therapy with noninvasive imaging is extremely valuable for better understanding the *in vivo* fate of nanomedicines, pharmacokinetics, target-site accumulation, and therapeutic efficacy.^39^ Furthermore, these nanomaterials’ outstanding biocompatibility, biodegradability, and multifunctional uses for biosensing, bioimaging, diagnostics, and therapies have expanded their applications in a vast range of biomedical applications.^96^ Following are the details of the organic cancer theranostics platform:

#### 5.1.1 Liposome Nanoparticles in Cancer Theranostics

Liposomes, a type of biomimetic nanoparticle, are undoubtedly the most well-known and adaptable lipid-based nanoweapon for cancer theranostics. These are generally made of concentric lipid bilayers that self-assemble around an aqueous core domain. They have proved to be effective nanocarriers for the delivery of a variety of drugs by encasing hydrophilic ones inside the liposomal aqueous core domain (or on the bilayer membrane surface) and hydrophobic ones inside the liposomal bilayer.^97^ These carriers offer many advantages, including biocompatibility, biodegradability, ease of synthesis, sustained release of the therapeutics, low toxicity, and the ability to incorporate both hydrophilic and hydrophobic chemotherapeutic compounds.^98,99^ Additionally, the liposome surfaces can be tailored for targeted cancer therapy.^100^ Liposomes can accumulate in cancerous tissues passively through the enhanced permeability and retention (EPR) effect and actively by specifically targeting a cancer cell or an angiogenic marker.^101^ Liposomal therapeutic agents with multimodality imaging make it highly impressive for individual monitoring of *in vivo* cancer and pharmacokinetics of therapeutic drugs. This platform also predicts the therapeutic efficacies of the drugs in combination with the useful information collected by imaging techniques.^102^ Numerous liposomal medications are now used in clinical trials or have received clinical approval due to their numerous benefits.^35,103,104^ Liposomes can transport small and large molecules and have also been investigated for the delivery of various diagnostic agents, such as ^64^Cu^105^ and ^14^C isotopes,^106^ quantum dots (QDs),^107^ gadolinium (Gd)-based contrast agents,^103^ etc. It is expected that liposomes as a theranostic tool for cancer patients will soon be applied in clinical trials.

#### 5.1.2 Lipid Nanoparticles in Cancer Therapy

Lipid nanoparticles (LNPs) remain one of the most promising platforms for cancer theranostics because of their biocompatibility and scalability.^108^ Lipidic nanocarriers have unique benefits that set them apart from other nanoformulations.^108^ Recent investigations have shown that lipidic theranostic nanomedicines are a promising and prospective strategy for increasing the efficacy of cancer treatment to a benchmark level.^42,109−111^ LNPs can penetrate the vascular endothelial gaps of tumors and deliver chemotherapy drugs to tumor tissue. In one of the studies, it has been found that DiR (DiIC_18_(7); 1,1′-dioctadecyl-3,3,3′,3′-tetramethylindotricarbocyanine iodide) dyes have the ability of enhanced tissue penetration owing to an elongated absorption wavelength for superior antitumor activity.^54^ The size of the nanocarrier strongly depends on the targeted organ and type of imaging. For the purpose of sensitive and specific tumor detection, Zhang et al.^112^ developed a fluorinated nanoemulsion. These nanoemulsions showed dramatically improved fluorescence imaging signals. Their theranostic method was extremely effective at identifying a particular type of tumor tracking the possible *in vivo* fate of the nanoemulsion and providing highly effective photodynamic therapy. Zheng et al. developed a nanoemulsion with a porphyrin shell that allows the encapsulation and stabilization of the oil core, resulting in a monodisperse nanostructure for imaging and phototherapy.^113^ A noninvasive and real-time monitoring of drug delivery for an orthopedic prostate tumor model was presented by Lin et al. by utilizing LNPs loaded with dye and siRNA.^114^ Liang et al. developed a theranostic nanoplatform based on a relatively smaller (<20 nm) iron oxide loaded with porphyrin-grafted lipid nanoparticles (Fe_3_O_4_@PGLNPs), demonstrating an excellent photodynamic effect against HT-29 cancer cells *in vitro*.^115^ LNPs are exclusively used in cancer theranostics agents for curing different types of tumors due to their negligible toxicity, multifunctional potential, and functionalization flexibility which helps them to cross different physiological barriers.^51,116−118^

#### 5.1.3 Protein Nanoparticles in Cancer Theranostics

Scientists extensively explored protein NPs because of their natural availability and compatibility with physiology. Proteins belong to the biological molecules with distinctive properties and perspectives, making them amenable to biomedicine and materials science.^119^ Due to the amphiphilicity nature of a protein, it interacts favorably with the drug and solvent, making it an ideal choice for NP preparation. The natural origin of a protein makes it biodegradable, metabolizable, and readily susceptible to changing surfaces to facilitate drug attachment and targeted ligand attachment. Albumin has been recognized as a potential carrier for delivering imaging/anticancer medicines to tumor microenvironments after the FDA’s clinical approval of Abraxane (paclitaxel bound to albumin).^120^ Patients had a better response rate with Abraxane than with conventional paclitaxel (Taxol), which increased progression-free survival with the least amount of side effects. Eventually, albumin was developed into a flexible delivery system for medications with poor water solubility, such as rapamycin (water solubility is 2.50 mg/mL).^121^ A clinical trial involving albumin-bound rapamycin (ABI-009) was conducted to treat nonhematologic cancers. There are many albumin-based NPs currently undergoing clinical studies.^122^ Human serum albumin (HSA) caps are among other intriguing possibilities since the human liver supplies a plentiful amount of the same (35–50 mg/mL).^123^ To build effective theranostics, HSA has been investigated as a natural transporter of superparamagnetic iron oxide, organic/inorganic oxides, IR780, IR825, and chlorin e6 (Ce6).^124^ NIR probes like IR825, indocyanine green (ICG), and IR780 are becoming more popular because of their ability to penetrate relatively deep tissue and their low autofluorescence interference. A recent publication noted that IR825 and gadolinium (Gd) were encapsulated in HSA to produce HAS-Gd-IR825 complexes for dual imaging-guided photothermal treatment (PTT) to prevent lymphatic metastases after surgery.^36^ Yu et al. developed gemcitabine and pheophorbide-a (P@) loaded human serum albumin (HSA) (P@-Gem-HSA) to create multifunctional nanoparticles for the treatment of lymphatic PDAC metastases.^125^

#### 5.1.4 Metal–Organic Frameworks in Cancer Theranostics

**Figure 6:**
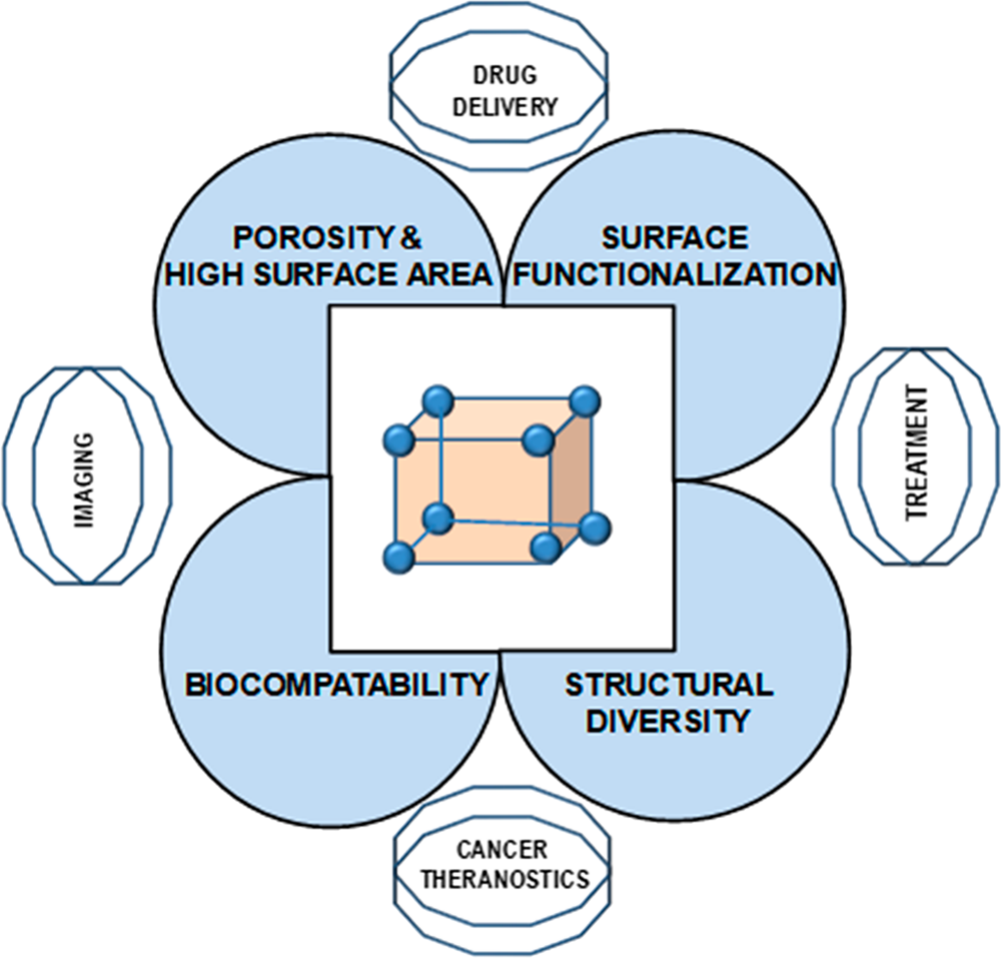
Schematic illustration of MOFs showing different applications for cancer diagnosis and treatment.

Metal–organic frameworks (MOFs), a fascinating and intriguing class of porous hybrid coordination polymers with metal ions or ion clusters serving as nodes and organic ligands serving as linkers, have been synthesized and utilized for a variety of applications, including gas storage, catalysis, sensing, biomedical applications, and cancer theranostics addressing the drawbacks of conventional cancer treatment or the inability of therapeutic drugs to target the tumor sites without damage to healthy tissues and organs. In order to synthesize structurally diverse MOFs (specific physical and chemical properties), metal (such as d- and f-block elements) and organic linkers (such as carboxylates and nitrogen heterocycles) can be varied, which leads to the synthesis of thousands of MOFs with distinctive characteristics.^126^ The development of nanoscale MOFs (nMOFs) presented potential applications in photodynamic therapy (PDT), drug delivery, and imaging and has led to considerable contemplation of their potential as therapeutic platforms in oncology and other fields of medicine, as shown in Figure 6. Their remarkable characteristics, which include easy surface functionalization, structural diversity, high surface area, enormous porosity, tunable energy gap, tailored synthesis, excellent biocompatibility, and a variety of physicochemical properties, make MOFs suitable candidates for cancer theranostics.^127−129^ A major reason for the failure of conventional cancer treatment is the inability of therapeutic drugs to reach the target tumor sites without damaging healthy tissues and organs. Due to their exceptional qualities, MOFs not only enhance the outcomes of conventional therapies like radiation therapy (RT) and chemotherapy but also benefit the recently developed phototherapy methods.^130−132^

Moreover, MOFs are suitable materials for imaging-guided cancer theranostics, as they can codeliver various bioactive substances, including medications, enzymes, genes, and gases.^133^ Zn-based MOFs have also been reported as nanocarriers. Wang et al. developed a chiral Zn-based MOF using zinc ions and achiral 5,5′,5″-(1,3,5-triazine-2,4,6-triyl)tris(azanediyl)triisophthalate (TATAT) ligands for the delivery of the anticancer medication 5-fluorouracil (5-FU). The experimental results indicated the high drug loading capacity and slow release of the loaded drug, with a complete delivery time of about 1 week.^134^ Gao et al. reported a multifunctional tumor targeting MOF nanocomposite with fluorescence (FL) imaging, MRI, and controlled drug release for cancer therapy. The targeting group folic acid (FA) and 5-fluorouracil agent were decorated on the surface of 5-FU-loaded Fe-MIL-53-NH_2_ as an outer layer through an amidation reaction to give Fe-MIL-53-NH_2_-FA-5-FAM/5-FU.^135^ In this approach, Fe-MIL-53-NH_2_ was utilized to encapsulate the drug and magnetic resonance imaging feature.

Liu and co-workers^136^ have synthesized UiO-66-NH_2_ with controlled particle sizes of 20–200 nm. Later the MOF was modified with FA and the fluorescence imaging agent 5-carboxyfluorescein (5-FAM) known as UiO-66-NH_2_-FA-5-FAM/5-FU, a multifunctional theranostic nanoplatform with FL imaging. The *in vivo* studies indicated that UiO-66-NH_2_-FA-5-FAM/5-FU could be accumulated in the tumor and display more vital antitumor efficiency due to the long-term drug release. In one study, Cherkasov et al. synthesized an MOF through antibodies to selectively absorb HER2/neu-positive cancer cells.^137^ A biomimetic MOF, Hf-DBP-Fe, was synthesized for effective cancer therapy utilizing a synergistic combination of radiation and an immune checkpoint blockade.^138^ Chen et al. synthesized folic acid modified hafnium-based manganoporphyrin metal–organic framework nanoparticles (MnTCPP-Hf-FA MOF NPs) to enhance radiotherapy and prevent postoperative recurrence.^139^Table 1 shows some representative MOFs for cancer therapy.

**Table 1:** Some Common MOFs for Cancer Therapy

MOF	active unit	therapeutic method	model	administration type	ref
Hf-DBB-Ru	Hf and Ru	radiotherapy	colorectal tumors in mouse models/MC38 tumor bearing C57BL/6 mice	intratumoral	(140)
W18@Hf12-DBB-Ir	Hf12 (SBUs), Ir bridging ligands, W-polyoxometalates	radiotherapy	murine colorectal adenocarcinoma models of MC38 tumor bearing C57BL/6 mice and CT26 tumor bearing BALB/c mice	intravenously	(141)
Hf-DBP-Fe	Hf, H_2_DBP, Fe	radiotherapy	MC38 cells	intraperitoneal	(138)
MnTCPP-Hf-FA MOF NPs	TCPP-Hf, Mn	radiotherapy	B16-F10 cells, melanomatumor	intravenous	(139)
DOX loaded MOFs	Zr^4+^ metal–organic framework nanoparticles, DOX	chemotherapy	MDA-MB-231 breast cancer cells	–	(142)
Fe-MIL-53-NH_2_-FA-5-FAM/5-FU	5-fluorouracil, Fe, BDC-NH_2_	chemotherapy	MGC-803 and HASMC, mice bearing glioblastoma	intratumoral injection	(135)
Fe_3_O_4_ /IRMOF-3/FA	Fe_3_O_4_, Zn(NO_3_)_2_·6H_2_O, NH_2_-H_2_BDC	chemotherapy	HeLa cells and normal NIH3T3 cells	–	(143)
Zr-UiO-66	Zr-UiO-66, DOX	chemotherapy	BALB/c mice, breast cancer cells and L929 fibroblasts	intravenous injection	(144)
DOx/FA/CDs/IRMOF-3	DOX, CDs, IRMOF-3	chemotherapy	L929 (murine fibroblast) and human cervix adenocarcinoma (HeLa) cell lines	–	(32)
fluorescein/ZIF-8	2-methyl imidazolate and zinc ions, fluorescein	chemotherapy	MCF-7 cell	–	(49)
UiO-66 and UiO-67	terephthalic acid (BDC), ZrCl_4_, 4,4′-biphenyldicarboxylic acid (BPDC)	chemotherapy	HSC-3 (human oral squamous carcinoma) and U-87 MG (human glioblastoma grade IV; astrocytoma)	–	(145)
selenium-polymer@ZIF8	2-methylimidazole, DOX@PZn(NO_3_)_2_·6H_2_O	chemotherapy	MDAMB-231 cells breast carcinoma cell line (MDA-MB-231)	–	(146)
cisplatin/doxorubicin/NMOF	cisplatin (14.4 wt %) and doxorubicin Zn^II^ (*p*-phenyleneethynylene) (OPE) dicarboxylate linker	chemotherapy	HeLa cells	–	(52)
Ti-TBPMOF	Ti–oxo chain (SBUs), 5,10,15,20-tetra(*p*-benzoato)porphyrin (TBP) ligands, Ti^3+^	photodynamic therapy	CT26 tumor bearing BALB/c mice	intratumorally	(147)
porphyrinic Zr-MOF	porphyrinic Zr metal–organic framework, MnO_2_, apatinib	photodynamic therapy	4T1 cells or mouse macrophage RAW 264.7 cells	subcutaneous and intravenous	(148)
Zr-TBB MOF	Zr, 5,10,15,20-tetra(*p*-benzoato)bacteriochlorin (TBB) ligands	photodynamic therapy	breast and colon cancers, 4T1 murine breast carcinoma cells, subcutaneous 4T1-bearing BALB/c mice and murine colon carcinoma MC38-bearing C57Bl/6 mice	subcutaneous	(149)
ZnP@Hf-QC	zinc phthalocyanine, HfCl_4_, H_2_QC	photodynamic therapy	CT26 cells, CT26 tumors on BALB/c mice, and MC38 tumors on C57BL/6 mice	subcutaneous	(150)
MOF199	Cu(II) carboxylate Ce, benzene-1,3,5-tricarboxylate (BTC), Cu(NO_3_)_2_	photodynamic therapy	HepG2 cells and NIH-3T3, HepG2 cells, and 3T3	intravenous injection	(151)

### 5.2 Carbon-Based Nanomaterials in Cancer Theranostics

One of the most promising fields of biomedical sciences with the fastest growth is nanotechnology, which has been cleverly applied to unravel various biological challenges.^35^ In recent years, carbon-based nanomaterials have significantly increased the detection and treatment of cancer and neurodegenerative diseases.^152,153^ Various research groups have concentrated on the development of carbon-based nanomaterials such as fullerene, carbon nanotubes, graphene, and derivatives for biomedical applications, which has opened the way for their application in the emerging field of cancer theranostics.^154,155^ The carbon-based nanomaterials exhibit several extraordinary properties, such as high surface area, tunable pore structure, and nonreactive and easy surface functionalization, making them suitable for their biological application, in particular, for cancer diagnosis, which in turns opens up a new avenue for improved therapeutic strategies.^156−158^ In addition to this, these nanomaterials possess outstanding biocompatibility, biodegradability, and multifunctional uses for biosensing, bioimaging, diagnostics, and therapies which have boosted their potential for biomedical applications.^159−161^

#### 5.2.1 Carbon Nanotubes

In one of the studies, carbon nanotubes (CNTs) have been shown to be a promising material for drug delivery. In this study, CNTs are loaded with ginsenoside Rg3 and fabricated Rg3-CNT, and the effect was studied on triple-negative breast cancer (TNBC). This study has established that Rg3-CNT is a potential therapeutic strategy for the immunotherapy of TNBC.^162^ M13 phage functionalized CNT (M13-CNT) was explored for the fluorescence imaging of targeted tumors even at low concentrations.^163^ In another work, a targeted M13 virus stabilized CNT probe was utilized for the detection of the human ovarian tumor by Ghosh et al. with a high signal-to-noise ratio. Results were compared with those with visible and NIR dyes, and it was found that the M13 virus stabilized CNT probe can detect a tumor in the submillimeter range.^164^ A fluorescence imaging system for ovarian cancer was developed by Ceppi et al. utilizing single-walled carbon nanotubes (SWCNTs) conjugated to M13 bacteriophage, which carries a peptide-specific protein.^165^ The developed system helps in intraoperative tumor debulking in real time. There was an improved survival rate in animals when treated with CNT fluorescence image surgery compared to conventional surgery. Lee and colleagues developed CNTs coupled with a platelet-derived aptamer, and the result showed a significant change in NIR fluorescence due to conformal aptamer change.^365^ Zhang et al. synthesized a nanocomposite comprising CNTs and CDs for dual-modal imaging of cancer cells.^166^ It was observed that the drug loading capabilities of CNTs depend on several factors, including the material’s nature, the medium’s pH, the free sites of the material, and the nature of the drug. Due to their substantial surface areas, SWCNTs have demonstrated a higher drug-loading capacity than MWCNTs.^167^ Peptides having aromatic content showed high binding affinity toward CNTs due to π–π interaction.^168^ Yang et al. proved that the functionalized SWCNTs as DOX carriers are useful for treating MCF-7 cells. Results demonstrated that, when compared to CNT-COOH and CNT-PEG, SWCNT-PEG-PEI had the most substantial antitumor impact and drug delivery capacity. Fluorescence-based research and flow cytometry investigations show that SWCNT-PEG-PEI is internalized more readily, promoting the apoptosis that leads to tumor cell death due to increased dispersibility and a stronger affinity for cancer cells.^169^ CNTs are exclusively used for the pH-responsive release of cancer drugs as they are very susceptible to the acidic environment, making them a promising candidate for drug therapy.^170^ Lu et al. functionalized CNTs with poly(acrylic acid) and used them for the FA for DOX drug loading. It has been found that DOX-loaded CNTs showed higher efficiency when compared to free DOX; this could be due to hydrogen bonding and π–π stacking. The developed platform serves as an efficient tool for cancer theranostics.^171^

#### 5.2.2 Carbon Dots

Owing to their small sizes, carbon dots (CDs) are particularly promising since they can target cancerous tumors with an increased permeability and retention (EPR) impact. The urinary system can readily eliminate CDs to reduce *in vivo* toxicity. Additionally, CDs have shown to be a unique platform for delivering different therapeutic agents. CDs are widely used for various applications such as chemotherapy, photodynamic therapy (PDT), photothermal therapy (PTT), gene therapy, and radiation therapy.^172−176^

The quantitative cellular accumulation of free CDs and CDs with targeting ligands on cancerous (MDAMB and A-549) and healthy (MDCK) cells showed that diseased cells were more effectively treated than healthy cells. The ligand-attached CDs that ingested cancer cells demonstrated target-selective endocytosis via receptor-mediated therapy. Additionally, the tumor’s uptake of nanosized CDs may be significantly influenced by the enhanced permeability and retention effect.^177−179^ Photosensitizing properties of CDs have been extensively explored for NIR light triggered photodynamic therapy alone or in combination with photosensitizing agents such as photoporphyrin, zinc phthalocyanine, etc., which are responsible for the production of reactive oxygen species (ROS) for the treatment of cancer cells.^180−184^

The targeted cancer cells can be hyperthermally killed by NIR-responsive photothermal treatments, like PDT. Numerous carbon-based nanomaterials tend to absorb NIR light from the electromagnetic spectrum and transform it into heat, which thermally kills cancerous cells.^185−187^ For example, under various excitation wavelengths, carbonized polydopamine has shown multiple fluorescence emissions along with NIR-responsive photothermal conversion and heat, which is responsible for the destruction of cancerous cells. CD-based hybrid systems are extensively explored for PDT, PTT, and pH- or NIR-responsive drug release all at once. Synergistic cancer therapy is made possible by such a multipurpose intelligent delivery system.^188^ For instance, CD nanogels with integrated PEG–chitosan have demonstrated PTT against tumor cells and dual pH- and NIR-light-responsive drug release.^184^ Mauro et al. developed an efficient protocol for one-pot synthesis of N, S doped CDs having a high NIR photothermal conversion efficiency and efficient ROS production in the cancer cell.^189^ S-CD triggers more ROS generation in cancer cells when compared to a healthy cell. The NIR laser is responsible to enhance the oxidative stress in cancer cells at a moderate power density and open up a range of possibilities for real biomedical applications. In another work,^190^ NIR-responsive core–shell hybrid nanocomposite as a smart theranostic platform based on hyaluronic acid–PLA and hydrophobic carbon dots (HA-*g*-PLA/HCDs) was designed to selectively recognize cancer cells overexpressing CD44 receptors and NIR-triggered chemo-phototherapy of solid tumors.

Moreover, combining the anticancer drug with NIR-triggered photothermal treatment may utilize the potential benefits of carbon dots, which may be overcome with specific resistance to apoptosis eliciting alternative RCD routes such as necroptosis. Nicosia et al. developed a hybrid material CD bearing biotin and a high amount of irinotecan (CDs-PEG-BT/IT) for controlled release and imaging of MDA-MB231 and MCF-7 cancer cells and killing them by photothermal and chemotherapeutic means.^191^ The results indicate that CDs-PEG-BT/IT is a safe and potentially effective candidate as a theranostic agent in IG-PTT of breast cancer. Geng et al. have synthesized nitrogen and oxygen codoped CDs with an excellent optical response to NIR lasers.^192^ These synthesized doped CDs showed excellent therapeutic efficacy and bioimaging ability with 100% tumor destruction without affecting the healthy cell.

CDs were also explored for the bioimaging and tracking of stem cells through endocytosis. Due to the nanosize, physicochemical properties, and surface charge of the nanoparticles, CDs can penetrate stem cells. They do not affect cell differentiation and expression of the specific marker. The rational design of CDs is anticipated to supplement the currently used fluorescence probes for biomedical applications.^193^

#### 5.2.3 Graphene

Graphene and its derivatives have been explored for biological and biomedical applications owing to their high surface areas, easy functionalization, and biocompatibility which render them for high drug loading capability and make them potential candidates as drug carriers, in imaging, and in drug delivery systems.^194,195^

Chen et al. have synthesized an rGO/PEG/ICG nanosystem, which is made up of the functionalization of reduced graphene oxide (rGO) with polyethylene glycol (PEG) and Indocyanine Green (ICG) and later used as a dual-modality contrast agent.^196^ It was observed that the rGO/PEG/ICG nanosystem was sustained for a longer time in *in vivo* and was capable of targeting tumors. Mirrahimi et al. used GO for anchoring superparamagnetic iron oxides (SPIOs) and AuNPs adapted with phase-change material (PCM) for stimulus-based drug release.^197^ The study also reveals that the synergistic effect of all, like NIR absorbance of GO, MRI contrast of SPIOs, AuNPs for X-ray attenuation, and PCM for thermosensitive features, make this platform ideally suited for synergistic thermo-chemotherapy in a controllable drug release.^197^ Graphene has the potential for use in PET, imaging, and dual-modality MRI/fluorescence imaging.^198−200^ A novel platform consisting of GO with PEGylated and oxidized sodium alginate was used to load the anticancer drug paclitaxel to get a synergistic chemotherapy/PTT/PDT effect.^201^ Yang et al. bound a molecular beacon (MB) on the surface of GO, which resulted in the enhanced fluorescence quenching of cy5 when compared to the self-quenching effect.^202^ The nanocomplex bound to miRNA-21, and the fluorescence response of cy5 was restored. With this technique, the fluorescence intensity was enhanced while the imaging’s fluorescence background was diminished. The synthesized nanocomplexes showed excellent imaging capability for numerous tumor cells, as shown by *in vitro* and *in vivo* studies, suggesting that GO-based nanomaterials had promising potential in cancer diagnostics. Graphene was also explored for breast tumor PTT and photoacoustic imaging (PAI). In this context, polydopamine (PDA) was bound to rGO, and then the diagnostic reagent ICG was loaded for PTT.^203^ It was found that PDA improved the nanosystem’s water solubility and biocompatibility, which in turn quenched rGO’s fluorescence and improved the effectiveness of PAI. The ICG-PDA-rGO was processed with higher sensitivity and PAI ability than other control groups, according to *in vivo* imaging investigations. The endogenous contrast agent hemoglobin also allowed for the clear observation of the vascular tissue in tumor tissue. The aforementioned findings suggested that rGO-based nanomaterials could be employed in PAI for tumor diagnostics as well as PTT to increase the effectiveness of cancer treatment. Graphene quantum dots (GQDs) show strong absorption in the NIR-II region. Liu et al. reported 9T-GQDs as a breast, cervical, and lung cancer imaging tool utilizing phenol as a precursor.^204^ The photoluminescence (PL) quantum yield of the 9T-GQDs was 16.67%, almost 1.8 times more than that of ordinary GQDs. The synthesized 9T-GQDs are able to reach the cytoplasm of the 4T1 cancer cell by penetration of the cell membrane to induce continuous fluorescence.

#### 5.2.4 Fullerene

A fullerene is a third allotrope of carbon with fused rings of five to seven carbon atoms joined by single and double bonds to form a closed or partially closed mesh.^205^ Fullerene exhibits a unique physicochemical characteristic, making it a promising candidate for several industrial and medical applications.^206^ Pristine fullerenes are insoluble in water, limiting their use in biological applications. The surface modification of fullerenes has opened up a new horizon for pharmaceutical and biomedical applications due to their water solubility after modification.^207^ Furthermore, fullerenes have drawn much interest in biomedical applications due to their small diameters, large specific surface areas, and high reactivity.^207^ Many water-soluble fullerene derivatives (C_70_, C_80_, C_94_) with various carbon atoms have also been studied to utilize their small sizes, shapes, and molecular topologies for various biological applications.^208−210^ For the cancer theranostics application, fullerenes can be functionalized to make them with distinct physicochemical characteristics such as biocompatibility and water solubility. Additionally, the fullerene cage functions as a unique 3D scaffold and may be utilized to attach a variety of drugs to it covalently.^211−213^

Fullerene is a promising nanomaterial for cancer imaging, PDT, and photo/thermoacoustic-assisted theranostics due to its intrinsic optical and thermodynamic qualities. Due to their distinctive physical–chemical characteristics, fullerenes have attracted a lot of attention in the field of cancer theranostics.^214^ Notably, fullerene can act as a potent antineoplastic agent due to its anticancer action and sensitization effect on cancer cells.^215^ Fullerene also acts as a free radical scavenger and can be employed as an antioxidant.^215^ It is interesting to note that metal atoms can also be added to fullerene to create metallofullerene, which inherits the combined properties of the carbon cages and internal metal and has promising potential for use as contrasts in magnetic resonance imaging (MRI), X-ray, radiotracers, and anticancer agents.^216^ Though with chemical modification the fullerene can be tailored for specific applications for cancer, there are some intrinsic challenges associated with fullerenes such as intrinsic toxicity and solubility in water. In this context, various research groups tried numerous approaches such as modification of fullerene with water-soluble functional groups (hydroxyl, carboxyl, and amino groups)/polymers or grafting of the biocompatible molecule through covalent bonding.^215^ Hence, owing to the unique advantages of biocompatible fullerene, it has been extensively explored as a potential platform for targeted drug delivery.^217^ To cure pancreatic cancer, Serda et al. created Sweet-C_60_ (highly water-soluble hexakis-glucosamine fullerene derivative), a novel targeted anticancer drug that mostly accumulated in the nucleus of pancreatic stellate cells (PSCs).^218^ It was observed that a glycoconjugate of fullerene could improve the cancer-targeting characteristics since the proliferation of tumors required the energy associated with glucose metabolism.

Wang et al. synthesized an amphiphilic fullerene derivative (C_60_-Dex-NH_2_) to deliver siRNA into cancerous cells to address the lysosomal degradation of RNAi.^219^ The results showed that the synthesized fullerene derivative promoted the lysosomal entrapment and caused the destruction of the lysosomal membrane by triggering controllable ROS under visible light irradiation. Through binding to the MYH9 protein, Zhou et al. created a class of C_70_ fullerene derivatives (C_70_-EDA) modified with multiple ethylenediamine (EDA) moieties that might prevent cancer cell migration, alter intracellular MYH9 distribution, and prevent EMT.^220^ C_70_-EDA treatment inhibited cancer cell migration and reversed the EMT process in A549 cells *in vitro*. Li et al. modified gadofullerene with β-alanine (GF-Ala) to develop a strategy against tumor immunity.^221^ This study revealed that immunosuppressive TME (ITM) is crucial for successful immunotherapy. This strategy could induce macrophages to exert an inhibitory tumor growth to convert to tumor-supportive M1 type from M2 type. From the above discussion, it can be concluded that judicial modification of fullerene is required to explore the excellent biocompatibility and significant antitumor activity, which can open up the horizon of materials for the broad application prospects in cancer therapy.

Table 2 summarizes the use of graphene and its derivatives for cancer imaging and theranostic applications.

**Table 2:** Graphene and Its Derivative Based Nanosystems for Cancer Imaging and Theranostics

system	cancer cell	experimental model (*in vitro*/*in vivo*)	main finding	ref
graphene oxide	murine lung metastasis model of breast cancer	*in vitro*/*in vivo*	Enhanced drug delivery efficiency in cbgLuc-MDA-MB-231 metastatic sites was demonstrated. It can serve as a useful tool for early metastasis detection and targeted delivery of therapeutics.	(222)
	B16F0 melanoma tumors using a mouse model	*in vitro*/*in vivo*	This work shows a nanocomposite as a theranostic nanomedicine for fluorescent imaging and combined nanomaterial-mediated photodynamic therapeutic and photothermal therapy for clinical cancer treatments.	(200)
	Bel-7402, SMMC-7721, HepG2 cell line hepatocellular carcinoma	*in vitro*	This work provides a multifunctional drug delivery system that has the ability to target hepatocarcinoma cells, is pH-responsive, and can be efficiently loaded with a number of therapeutic agents for biomedical applications.	(223)
	MCF-7 cells	*in vitro*	Photoresponsive ICG-loaded HArGO nanosheets could serve as a potential theranostic nanoplatform for image-guided and synergistic photothermal antitumor therapy.	(224)
	KB cell bearing nude mice	*in vitro*/*in vivo*	This provides an effective tool to visualize intracellular low-level miRNAs and to help in understanding the role of miRNAs in cellular processes.	(225)
	PC-3 cells prostate cancer	*in vitro*	This work evaluated interactions between halogenated amino acid tagged fluorescent NDIs and flat aromatic carbon materials.	(226)
rGO-MnFe_2_O_4_-PEG	4T1 tumor	*in vitro*/*in vivo*	Multimodal imaging using nanomaterials in a single platform can provide exact information including the tumor location and size. This study also promotes the biomedical applications of nanographene based nanocomposites.	(227)
CoFe_2_O_4_/GO cobalt ferrite/graphene oxide	cancer cell	*in vitro*	The prepared material showed great potential as an effective multifunctional nanoplatform for magnetic resonance imaging and controlled drug delivery for simultaneous cancer diagnosis and chemotherapy.	(228)
GO/ZnFe_2_O_4_/UCNP graphene oxide/Zn ferrite/upconversion luminescence nanoparticles	HeLa cell	*in vitro*/*in vivo*	Diagnosis to therapy realized the integration of imaging and high antitumor efficiency. It provides a feasible strategy to solve the main problems in current light-triggered theranostics.	(229)
GO-CD/Fe@C graphene oxide–cyclodextrin, carbon-coated iron NPs	MDA-MB-231 breast cancer cells	*in vitro*	The material was used for potential magnetic-directed drug delivery and as a diagnostic agent. The finding highlights the multifunctional GO-CD/Fe@C nanohybrid for magnetic sensing anticancer drug delivery to tumor cells.	(230)
MNP/GO/chitosan chitosan-grafted graphene oxide/magnetic nanoparticle	CD44-expressing breast cancer cells, MCF-7 human breast cancer, and L-929 mouse fibroblast cells	*in vitro*	This work highlighted cancer treatment for transformative theranostic technologies combining imaging with drug delivery.	(231)
fullerene	HCT 116 colon cancer	*in vitro*	Fullerene was used for high-resolution fluorescent imaging of tumor sites *in vivo* and resulted in a significant regression of HCT-116 tumors.	(232)
fullerene derivative (C_60_-Dex-NH_2_)	MDA-MB-231 cells	*in vitro*/*in vivo*	This work presented a novel photosensitive siRNA delivery carrier, C_60_-Dex-NH_2_. The C_60_-Dex-NH_2_/siRNA complex destroyed the endolysosomal membrane via the controllable generation of ROS when exposed to visible light, which enhanced the gene silencing efficiency of the siRNA *in vitro* and *in vivo*.	(233)
C_70_ fullerene derivatives (C_70_-EDA)	A549 cells	*in vitro*	This work provides a precise biological target and new strategies for fullerene applications in cancer therapy.	(220)
gadofullerene (Gd@C82) with β-alanines (GF-Ala)	RAW 264.7 cells, 4T1 and A549 cancer cell	*in vitro*	This study provides an effective immunomodulation strategy using gadofullerene nanoparticles by rebuilding ITM and synergizing immune checkpoint blockade therapy.	(221)
fullerene modified with diadduct malonic acid, micelles	HeLa cells, S180 tumor bearing mouse models	*in vitro*/*in vivo*	This study suggested the tremendous promise of DMA-C_60_ as a carrier material of MC and significant advantages in a combination of chemo-phototherapy of some tumors.	(234)
fullerene modified with distearoyl-*sn*-glycero-3-phosphoethanolamine, polyethylene glycol, Asn-Gly-Arg (NGR)	4T1 cells (mouse breast cancer cell line)	*in vitro*/*in vivo*	To address the problem of traditional drug delivery such as unexpected drug release during circulation and the sluggish release of drug in the target site, an “off–on” type drug delivery system with precise control was developed in this study.	(235)
fullerene with Cremophor EL micelles	human cervical HeLa cancer cells	*in vitro*	This study reported the synthesis and anticancer photodynamic properties of two new decacationic fullerene (LC14) and red light harvesting antenna–fullerene conjugated monoadduct (LC15) derivatives.	(236)
SWNT	KB oral epithelial carcinoma	*in vitro*	This work presents a facile approach to synthesizing water-soluble noble metal-coated SWNTs with a strong SERS effect for Raman spectroscopic imaging of biological samples. The nanocomposite can be used as an optical theranostic probe for cancer imaging and therapy.	(237)
	MCF-7 breast cancer	*in vitro*/*in vivo*	In this work, an aspargine–glycine–arginine peptide-modified SWCNT system was developed by a noncovalent approach and loaded with the doxorubicin and MRI contrast agent gadolinium diethylenetriamine pentaacetic acid.	(238)
	human breast cancer	*in vitro*	In the present study, the efficacy of multiscale photoacoustic microscopy was investigated to detect, map, and quantify trace amounts (ng to μg) of SWCNTs in a variety of histological tissue specimens consisting of cancer and benign tissue biopsies.	(239)
	4T1 breast cancer	*in vitro*	This work presented a new type of theranostic platform based on SWNTs coated with a shell of polydopamine followed by polyethylene glycol.	(240)
	MDA-MB-468 breast cancer	*in vitro*/*in vivo*	In this work, SWNTs dispersed in sodium cholate with the biocompatibility of SWNTs dispersed in PL–PEG and establishes the use of bright biocompatible SWNTs as versatile *in vivo* NIR photoluminescence imaging agents for live animals.	(241)
MWNT	B16F10 melanoma	*in vitro*/*in vivo*	This study suggests the utilization of MWNTs for the codelivery of tumor-derived antigen, CpG, and αCD40 for efficient tumor eradication.	(242)
	A549 lung cancer	*in vitro*/*in vivo*	This work presents a dual-targeting strategy that improves the delivery performance of MWNT and opens a new avenue for RAS-related lung cancer therapy.	(243)
	MCF-7 human breast cell lines	*in vitro*	In this work, novel biomaterials utilizing water-soluble chitosan (CS), phycocyanin, MWCNTS were prepared and characterized with the potential for PDT and PTT.	(244)
MWNTs/gemcitabine (Ge)/lentinan	MCF-7 cells	*in vitro*/*in vivo*	Multiwalled carbon nanotubes (MWNTs)/gemcitabine (Ge)/lentinan) three-component anticancer composite was prepared and shown to be a promising candidate for cancer therapy in the combination of chemotherapy and photothermal therapy.	(245)
MWCNTs-TAM-LEN lentinan tamoxifen	MCF-7 cells	*in vitro*/*in vivo*	A lentinan (LEN) functionalized multiwalled carbon nanotubes (MWCNTs) drug delivery system, using tamoxifen (TAM), was developed. This system possessed good stability, water dispersibility, and extraordinary photothermal properties.	(246)

### 5.3 Engineered Inorganic Nanoparticles in Cancer Theranostics

Due to their unique physicochemical characteristics, inorganic nanoparticles such as platinum, gold, silica, palladium, silver, iron oxides, zinc oxide, and rare earth oxides have been widely used for a number of biomedical applications including cancer theranostics, nucleic acid delivery, bioimaging, drug administration, and biosensing.^61,247,248^

Green chemistry is a promising approach that can be used for the natural design of metallic or nonmetallic nanoparticles.^249^ Many advantages are associated with this technique, including their eco-friendly setups, which ignore harmful chemicals, simplicity, robustness, time savings, and use of safe solvents, especially water.^250^ This green approach uses bioreducing agents comprising plant extracts, bacteria, algae, etc., in place of hazardous chemical catalysts. Because of these benefits, such safe, natural nanoparticles are highly desirable for cancer theranostics.^251^

In many parts of the world, various research teams are utilizing nanoparticles of silver and gold synthesized by a biological process that can work as one of the ideal candidates for cancer theranostics applications. AuNPs and AgNPs produced through biosynthesis have the potential to be used in the *in vitro* and *in vivo* delivery of anticancer medications.^252^ Mukherjee et al. demonstrated the transport of doxorubicin (DOX) to an *in vitro* and *in vivo* mouse melanoma tumor model utilizing biosynthesized gold nanoparticles (b-AuNPs) made from the aqueous leaf extract of *Peltophorum pterocarpum* popularly known as “yellow flame tree”.^253^ Outstandingly, these b-AuNPs were shown to be exceptionally biologically attuned when C57BL6/J mice were cured with b-AuNPs as compared to mice cured with (chemically designed) AuNPs subsequent to successive intraperitoneal injections of 10 mg/kg of body weight. In addition, melanoma tumor development in mice was significantly inhibited by b-AuNP conjugated DOX treatment compared to pristine DOX. In another study by Ganeshkumar et al., the anticancer medication 5-fluorouracil was administered to breast cancer cells in a targeted way utilizing folic acid linked b-AuNPs (made using *Punica granatum* fruit peel extract).^44^ These b-NPs were used as anticancer medicines because the bioresources that adhere to the nanosurface during the manufacturing process contain therapeutically active phytochemicals (isoflavone, flavonoids, polyphenols, taxol, etc.). As a result, b-NPs showed a distinct advantage over NPs created chemically. Mukherjee et al.^366^ demonstrated the 4-in-1 theranostic uses of biosynthesized silver nanoparticles employing a methanolic extract of *Olax scandens* leaves (biocompatible, anticancer, cell imaging, and antibacterial). Mukherjee et al.^254^ showed that b-AuNPs made from *Lantana montevidensis* leaf extract have antitumor action. The anticancer phytochemicals cirsilineol, apigenin, eupatorine, hispidulin, eupafolin, and β-caryophyllene are responsible for their therapeutic efficacy in *L. montevidensis* leaf extract. Fazal et al.^255^ exhibited the photothermal ablation capabilities of anisotropic b-AuNPs produced using cacao seed extract toward epidermoid carcinoma A431 cells grown *in vitro*. Wang et al.^256^ demonstrated fluorescence-based bioimaging of *in situ* b-AuNPs for identifying malignancies in cancer cells. Chloroauric acid (HAuCl_4_) was incubated with human leukemia cells (K562) and human hepatocarcinoma cells (HepG2), which produced green fluorescence. Noncancerous L02 (human embryo liver cell strand) cells did not have any fluorescence after incubating with HAuCl_4_, demonstrating the specificity of b-AuNP synthesis to cancerous cells. Following subcutaneous injection of HAuCl_4_ solution (10 mmol/L), the scientists further demonstrated the applicability of *in vivo* bioimaging to an *in vivo* xenograft tumor model of HepG2 or K562 cancer cells in BALB/c mice. The *in vivo* fluorescence showed high fluorescence surrounding the tumor even 72 h after HAuCl_4_ injection, showing the persistent fluorescence of *in vivo* biosynthesized b-AuNPs, which can be used for tumor diagnosis. AuNPs are extensively used in targeted drug delivery along with X-ray imaging, NIR imaging, fluorescence imaging and photoacoustic imaging as they have a tendency to accumulate in the tumor at the cellular level.^257^ Kotcherlakota et al.^258^ synthesized greener AuNPs using *Zinnia elegans* (ZE) plants. The synthesized AuNPs were tested for biocompatibility with the cancer cell lines using an MTT assay and were found to be biocompatible. This study has proven that AuZE NPs can be used in diagnostic imaging as these NPs are homogeneously distributed in the brain of mice without the ligand and exhibit strong fluorescence in the NIR region. AuNPs have great potential for diagnostic and theranostic tools due to their optical and chemical characteristics.^259^ The surface functionalization of these NPs makes them a potential nanovehicle for cancer treatments.^260^

Silver nanoparticles (AgNPs) are one of the most important and fascinating nanomaterials and have been employed as an antibacterial agent since ancient times. The use of AgNPs was also witnessed in World War I to treat bacterial infections. Owing to unique physiochemical and biological properties, AgNPs have attracted significant attention in cancer theranostics. AgNPs exhibit optical properties of surface plasmon resonance and can be tuned to the visible spectrum for lower detection limits. Austin et al. have captured the image of cancer cells (phagocytic activity) using light-scattering dark-field microscopy where AgNPs were incubated with oral squamous cell carcinoma (HSC-3).^261^*Moringa oleifera* aqueous stem bark extract and *Styrax benzoin* gum powder materials were explored for the greener synthesis of AgNPs and later used for the apoptosis of HeLa cells due to ROS production by AgNPs.^262,263^ In another study, Gurunathan et al. reported the preparation of AgNPs by using leaf extract of *Artemisia princeps*, which was used as a bioreductant, and AgNPs were found to be killing A549 lung carcinomas without any adverse effect on normal lung cell.^264^

Padinjarathil et al. have utilized a surfactant-free environmentally friendly technique for the synthesis of AgNPs, and it provides a potential theranostic platform with synergistic anticancer and immunomodulatory potential in the same platform.^265^ The AgNPs were synthesized from galactomannan with an average size of around 30 nm and a negative surface charge of 35.2 mV. In a work by Oves et al., AgNPs were synthesized using root extract of *Phoenix dactylifera*.^266^ FTIR, XRD, and UV–vis characterization studies were carried out to see the nature of synthesized NPs. The developed AgNPs were tested for antimicrobial and anticancer potential and destroyed cancer cells by arresting the cell cycle at sub-G1 and S phases. Biogenic silver nanoparticles (2–50 nm) were synthesized utilizing an extract of the latex of *Calotropis gigantean.* The results showed that the NPs are toxic to breast cancer cells, lymphoblastic leukemia (Jurkat cell lines), and ascites tumor cells (EAC cell lines) without producing cytotoxicity in mice and human lymphocytes.^267^ The results reveal that AgNPs could be a potential chemotherapeutic formulation for cancer therapy. AgNPs produce ROS that lead to damage to DNA, triggering apoptosis, and damage to the cancerous cells.^268^ This study also revealed that AgNPs affected the cells’ respiration and the vascular endothelial growth factor activity, which is responsible for angiogenesis. Dinparvar et al.^269^ used seed extract of *Cuminum cyminum* to synthesize bio-AgNPs, and efficacy was checked against the human breast cancer cell line MCF-7 and cancer cell line AU565. This study observed that chemically synthesized AgNPs exhibit toxicity while biologically synthesized AgNPs were far less cytotoxic and exhibited strong inhibitory effects against human breast cancer cells. Kumar et al.^270^ synthesized AgNPs using Andean mora leaf. Their efficacy was checked against Hep-G2 human liver cancer cells, and it was found that the synthesized AgNPs are useful for anticancer therapy and drug delivery.

Copper-based nanoparticles (Cu_2_O, CuO, Cu_2_S, CuS, Cu_2_Se, CuSe, etc.) have attracted the scientific community’s attention because of their biocompatibility and unique physicochemical properties.^271^ The synthesis of CuNPs mainly includes solvothermal/hydrothermal methods, colloidal synthesis, thermal decomposition methods, microwave-assisted synthesis, cationic exchange methods, and template-oriented synthesis methods.^272,273^ Zhao et al. have synthesized Ag_2–*x*_Cu_*x*_S quantum dots and characterized all-in-one theranostic nanomedicine photothermal therapy.^274^ The resultant materials show high photothermal conversion efficiency, and PTT can be performed under a low-power laser of 635 nm and demonstrates no long-term toxicity.

CuS nanocrystals (NCs) were grafted onto the surface of gelatin nanoparticles. These transformable nanoparticles, CuS@GNPs, were later used for the thermal ablation of human MDA-MB-23 tumors.^275^ The spatiotemporal multistage delivery behavior of CuS@GNPs within tumor tissue was observed *in vivo* and in real time by photoacoustic imaging. The multistage behavior of CuS@GNPs enhances photothermal ablation. The result indicated that the developed method may undergo enzyme-induced multistage administration for increased penetration and accumulation at the tumor site. Wang et al. have developed iron–copper codoped polyaniline (Fe–Cu@PANI) NPs for simultaneous imaging and photothermal therapy.^276^ In this work, glutathione (GSH) was explored for diagnosis and tumor microenvironment activated therapy. GSH is responsible for the reduction of Cu^2+^ ions and produces protonated PANI. This redox reaction induces a red shift from 615 to 815 nm. This study paves the way for numerous potential application prospects in the detection and treatment of cancer. For PDT, copper doped carbon dots (Cu-CDs) were prepared and utilized for cervical cancer and neuroblastoma. Cu-CDs generated ROS with a quantum yield of 0.36 after irradiating with LED light. The prepared Cu-CDs exhibited an effective tumor suppression effect on HeLa cells and SH-SY5Y 3D multicellular spheroids (MCs). Deng et al. have synthesized a series of copper nanoparticles with tunable acid dissociation constant (p*K*_a_) values from 5.2 to 6.2 for H_2_O_2_ self-supplying CDT.^277^ This study implies that lower p*K*_a_ value NPs exhibited better retention when compared to higher value ones. The catalytic ion produced by NPs converted self-supplied H_2_O_2_ into hydroxide free radical by the Fenton reaction, which permeates the lysosomal membrane and thus employs lysosome-mediated cell death to eliminate tumor cells.^277^

#### 5.3.1 Quantum Dots in Cancer Theranostics

Over the past few decades, researchers have paid significant attention to multimodal medication delivery systems. Due to their distinct physiochemical properties, quantum dots (QDs) are among the most effective tools utilized in theranostic applications for diagnosis and therapy.^278^ QDs are semiconductor crystals in the 2–10 nm nanoscale range and show emission from visible to near-infrared wavelengths along with outstanding light stability.^279,280^ The best QDs for cell labeling and cancer biomarker detection are those with intense photoluminescence and high molar extinction coefficient values. Symmetrical broad-absorption spectra and narrow-emission spectra distinguish QDs.^281,282^ However, due to the release of Cd ions and the production of reactive oxygen species, some concerns have been raised about the toxicity of QDs, particularly QDs containing Cd.^283^ In order to provide effective tumor targeting and prevent their escape into the systemic circulation, techniques have been devised to minimize their toxicity and improve their biocompatibility through hybridization with other moieties such as proteins, lipids, polymers, and polysaccharides.^284−287^

High-stability protein–QD nanohybrids in biological fluids are essential for bioimaging applications. QDs can combine with proteins by either physical trapping or chemical interaction.^288^ For instance, gemcitabine-loaded human serum albumin nanoparticles have successfully had graphene QDs attached to their surfaces (NPs).^289^ Nigam et al. reported hyaluronic acid and graphene QDs functionalized human serum albumin nanoparticles for bioimaging and targeted delivery of gemcitabine to pancreatic cancer.^289^ In a different work, spray-dried single bovine serum albumin (BSA) nanospheres were used to physically encapsulate several CdTe/CdS QDs of various sizes to create multifluorescent nanospheres,^290^ which can be achieved by altering the QDs’ size. When the QD-BSA nanospheres comprised a high molar ratio of QD:BSA, the fluorescence emission was reduced by 4% after being continuously irradiated (at 365 nm) for 1 h.^290^ These findings demonstrated the potential of QD-BSA nanospheres for long-term fluorescence observation in biomedical research fields. Song et al. synthesized a nanocomposite of phosphorus QDs with Bi@RP-PEG-DOX and bismuthene, which showed an effective platform for photothermal and photodynamic effects and controlled release of the drug in the presence of NIR-II irradiation.^291^ Zein–ZnS QD nanohybrids could be used to deliver drugs like 5-fluorouracil.^292^ Girija Aswathy et al. examined the impact of these nanohybrids on the viability of L929 and MCF-7 cancer cells to ascertain their biocompatibility.^292^ For zein-QDs and ordinary zein nanoparticles, the cell viability was found to be above 90 and 80%, respectively, which showed that they were compatible. Cell viability was considerably decreased when the cells were exposed to 5-FU-loaded zein-QDs. Furthermore, the zein–ZnS QD NPs’ fluorescence emission demonstrated that they had successfully entered the cells. Shao et al. synthesized biodegradable titanium nitride quantum dots (Ti_2_N QDs), which showed excellent photothermal conversion efficiency under NIR.^293^ The prepared material showed excellent biocompatibility, photoacoustic effect, and efficiency of photothermal therapy (PTT). Another study indicated multistage QD nanocarriers by joining silica-coated QDs to the surface of gelatin NPs to create 100 nm nanohybrids, among other intriguing methods to improve tumor penetration.^294^ In the tumor microenvironment the increased matrix of metalloproteinases dissolved the gelatin core after extravasations into the tumor tissue, producing incredibly tiny 10 nm QDs that effectively penetrated the tumor parenchyma. In general, utilizing proteins to hybridize QDs can be a viable method to extend their time in the body, boost their physical stability, improve their capacity to target tumors, and lessen their toxicity.

To enable nontoxic imaging, various polysaccharide types can be used to increase the safety and biocompatibility of QDs. Chitosan toxicity can be decreased by adding Mn-doped ZnS QDs (Mn:ZnS QDs), making folic acid functionalization possible.^295^ Bwatanglang et al. integrated the targeted drug delivery properties of folic acid (FA) with the imaging properties of Mn:ZnS QDs into a unified nanodelivery system.^295^ In order to create an FACS–Mn:ZnS nanocomposite, FA–chitosan conjugate (FACS) was first synthesized and electrostatically complexed with Mn:ZnS QDs. After 24 h, neither bare Mn:ZnS QDs nor FACS–Mn:ZnS (7–500 g/mL) displayed toxicity to cancer models of breast cells (MCF-7 and MDA-MB-231) or a healthy model of breast cells (MCF-10). Only a minor decrease in viability was seen in FACS–Mn:ZnS treated cells as compared to treatment with Mn:ZnS QDs when the QD concentration was increased from 62 to 500 g/mL. This marginal decrease in cell survival for the cells treated with FACS–Mn:ZnS may be attributed to the enhanced binding of the FA-coupled QDs to the cancer cell that expresses the folate receptor. Also, upon binding to the folate receptors expressed on the cancer cells, the FA-conjugated QDs increased the intensity of the cellular fluorescence.

Hyaluronic acid and QD pairing have been discovered to be an intriguing strategy for improved intracellular transport into liver cells, mediated by contact with CD44 receptors, permitting *in vivo* real-time observation.^296,297^ In another work, CdTe QD theranostic nanocapsules were coated with chondroitin sulfate followed by loading with rapamycin and celecoxib for cancer applications.^298^ To prevent chondroitin sulfate nanocapsules from being absorbed by healthy cells unintentionally, an exterior coating of cationic gelatin-coupled QDs was applied to them. Gelatin was degraded by matrix metalloproteinases at tumor locations, releasing both medication nanocapsules and quantum dots (QDs) into cancer cells for imaging.^299^ In a related experiment, lactoferrin, an iron-binding cationic protein, was used in place of gelatin to produce an on–off effect, where the fluorescence of QDs was first quenched by energy transfer and subsequently recovered upon bond cleavage in tumor cells.^299^ As a result, QD fluorescence was used to show how nanocapsules were localized both *in vitro* and *in vivo* within breast cancers. On the basis of the studies mentioned above, it is clear that combining QDs with polysaccharides can enhance their ability to target tumors by interacting with their receptors, which cancer cells tend to overexpress.^300^ This leads to an increase in the amount of QDs that accumulate at the tumor site. Table 3 summarizes the application of various nanomaterials in targeted cancer therapy and imaging.

**Table 3:** Representative Theranostic Nanomaterials and Their Applications

no.	type of nanomaterial	characteristics	application	cancer treatment	technique used	ref
1	lipid-based nanoparticles	colloidal carriers, biocompatible, biodegradable, and amphiphilic	nanocarriers for codelivery of hydrophilic and hydrophobic drugs controlled and modified drug release, preventing drug degradation	improving antitumor activities of several chemotherapeutic agents	ME cold dilution	(301)
2	protein nanoparticles	self-assembled supramolecular structures, biocompatible, biodegradable and low immunogenicity; one of the FDA-approved drugs; easy functionalization	cancer imaging and therapy	metastatic breast cancer	genetic recombination	(302)
3	viral nanoparticles	nanomaterials are derived naturally from plant viruses, bacteriophages, and mammalian viruses; noninfectious, biocompatible, and biodegradable	drug delivery, imaging, immunotherapy, theranostic applications, cancer vaccine, and immune modulators	cancer immunotherapy	computer-based technology	(303)
4	inorganic nanoparticles	SPR effect and photothermal effect with unique properties including optical, thermal, and electrical conductivity, potential magnetic and catalytic properties; some of the materials have issues with *in vivo* long-term circulation and potential toxicity	drug carrier, imaging, therapy, and functional coating drugs that lead to more effective antitumor activity	cancer imaging and therapy	photodynamic therapy and hyperthermia	(304)
5	bioinspired nanoparticles			cancer theranostics	fluorescence	(21)
6	ZnO QDs conjugated with gold NPs	porous structure, biocompatibility, biodegradability, and unique physicochemical properties	delivery platform, photodynamic therapy, photothermal therapy, imaging, PA imaging, radiotherapy, and phototherapy	tumor-targeted drug delivery	electrochemical	(305)
7	pectin–guar gum–zinc oxide (PECGG–ZnO) nanocomposite	porous structure, biocompatible, biodegradability, and unique physicochemical attributes	effective drug carrier for targeted and sustained delivery of various bioactive and chemotherapeutic anticancerous drugs; specific toxicity via generation of reactive oxygen	tumor-targeted drug delivery	precipitation	(306)
8	mesoporous ZnO nanofibers (ZnOnFs)	porous structure, biocompatible, biodegradability, and unique physicochemical attributes.	effective drug carrier for targeted and sustained delivery of various bioactive and chemotherapeutic anticancerous drugs; specific toxicity via generation of reactive oxygen	breast cancer	electrospinning	(307)
9	Mn-doped ZnS QDs	nonradiative decay, superior stability, such as high fluorescence intensity, long lifetime, and good resistance to photobleaching	delivery platform, photodynamic therapy, photothermal therapy, and imaging	chondrosarcoma	photodynamic therapy	(308)
10	nonmetallic nanomaterials	cost-effective, biocompatible, easy modification protecting drug against multidrug resistance	environmental friendliness, diminutive toxicity, and low cost, stimuli-responsive systems for the transport and delivery of materials, optical labeling or other detectable tracers, carrier, NIR fluorescent probe, and photothermal therapy	cancer theranostics	microemulsion	(309)
11	paclitaxel-loaded PLGA nanoparticles	biodegradable polymeric NPs used in medicine delivery systems and given approval by the FDA for its controlled and sustained release features	drug carriers and drug delivery, and ease of processing	anticancer therapy	ultrasound imaging	(310)
12	nanoscale metal–organic frameworks (nMOFs)	high surface areas, tunable pore size, crystallinity, thermal stability, and easy surface modification	effective cellular uptake, focused molecular targeting, smart nanodrug delivery devices, and drug-responsive behavior; extensive therapeutic stimuli and drug release	cancer immunotherapy	radiotherapy–radiodynamic therapy (RT–RDT) and chemodynamic therapy (CDT)	(311)
13	polylactide nanoconjugates	biodegradable and biocompatible polymer and easy to modify	suitable for usage as a nanoparticulate platform for administration of antigens and drugs	prostate cancer therapy	intravenous therapeutic strategy	(312)
14	DOX/ICG/BSA nanoparticles	large surface area to volume ratio, biocompatible	effective carriers for delivering ICG to tumor tissue, drug delivery	breast cancer therapy	photothermal therapy	(313)
15	dendrimer-based nanoparticles	polymer containing repetitive unit with a symmetric, almost spherical shape; the majority of polymers are not soluble in water; it is possible to make dendrimers water-soluble	high loading capacity for guest molecules; diagnosis using imaging techniques with low toxicity, low immunogenicity, and high permeability so that they can cross biological barriers	cancer therapy	MRI	(314)
16	carbon nanotubes	one-dimensional material, inert, stable, and biocompatible	drug carriers deliver various anticancer cancer agents; excellent mediators in phototherapy because of their inherent optical features	cancerous cells detection and drug delivery	photoacoustic imaging	(315)
17	porous nanomaterials (PNMs)	porous structure, easy modification and protecting drug against multidrug resistance, stable, and biocompatible	drug carrier	cancer immunotherapy	photothermal	(316)
18	gold nanoparticles	one-dimensional, SPR effect, and photothermal effect; strong photostability and photoluminescence	excellent plasmonic materials size adjustable energy regulation; immobilization of bioreceptors, improved analyte loading, strong catalytic characteristics	cancer therapy	photoacoustic imaging	(317)
19	plasmonic palladium (Pd) nanospheres coated with titanium dioxide shell	SPR effect and photothermal effect; strong photostability and photoluminescence	immobilization of bioreceptors, improved analyte loading, strong catalytic characteristics	cancer therapy	photothermal technique	(318)
20	silver nanoparticles	one-dimensional, unique physicochemical properties including optical, thermal, and electrical conductivities as well as their capability to combat viruses, fungi, and even bacteria	immobilization of bioreceptors, improved analyte loading, strong catalytic characteristics	antitumor agents	biomedical imaging	(319)
21	gold nanorods	one-dimensional, SPR effect and photothermal effect; high photostability and photoluminescence	excellent plasmonic materials size, adjustable energy regulation; immobilization of bioreceptors, improved analyte loading, strong catalytic properties	breast cancer therapy	photothermal	(320)
22	aptamer-based nanoparticles	aptamers are single-stranded DNAs or RNAs; highly selective recognition ability; good reproducibility, low toxicity, high stability, low molecular weight, and compact size	aptamers due to structure diversity have higher rates of tumor penetration, retention, and uniform dispersion, while the attachment process to the surface of nanomaterials is more amenable and repeatable	cancer therapy	cellular imaging	(321)
23	porphyrin-based inorganic nanoparticles	photosensitizer, tunable surface area, and biocompatibility	photodynamic therapy, cancer treatment and photothermal therapy as well as enhanced photodiagnosis	cancer treatment	photodynamic therapy	(322)
24	gold nanoparticles/graphene oxides	SPR effect, strong NIR light absorption and high surface area; high photostability and photoluminescence	immobilization of bioreceptors, improved analyte loading, strong catalytic properties; drug carriers, NIR florescence probes, PA imaging photothermal therapy	breast cancer therapy	near-infrared (NIR) light-activatable photothermal therapy	(323)
25	liposome-based nanoparticles	amphiphilic and good biocompatibility; however, easily oxidized, structurally unstable	nanocarriers for codelivery of hydrophobic and hydrophilic drugs	cancer therapy	chemo-immunotherapy (CIT)	(324)
26	mesoporous MnO_2_	porous structure, easy modification, and protecting drug against multidrug resistance; stable, biocompatible	drug carrier	breast cancer	photodynamic therapy	(325)
27	Fe_3_O_4_-based nanoparticles	magnetic properties and biocompatibility, high surface-to-volume ratio, and easy separation methodology	MRI imaging, hyperthermia treatment, drug delivery, superparamagnetism	targeted drug/gene delivery systems	chemotherapy	(326)
28	Ag–Au nanostructure	SPR effect and photothermal effect	PA imaging, Raman spectroscopy imaging radiotherapy, phototherapy, immobilization of bioreceptors, improved analyte loading	breast cancer therapy	near-infrared photothermal therapy	(327)
29	gold nanoparticles	SPR effect and photothermal effect; photostability and photoluminescence	immobilization of bioreceptors, improved analyte loading, strong catalytic properties	colorectal cancer therapy	chemotherapy	(328)
30	Fmoc-H/Zn^2+^/OMHEPzEOPP nanoparticles	photothermal conversion properties, high surface area, and biocompatible	exceptional superparamagnetic characteristics, photothermal therapy, photodynamic therapy, and enhanced drug delivery systems (DDSs)	cervical cancer therapy	near-infrared nanocomposite photosensitizer	(329)
31	persistent luminescence nanoparticles	unique optical materials, easily doped or modified with elements, hollow or mesoporous structures, and versatile surface functionality	optical imaging of tumors, high sensitivity, high penetration depth, fluorescence-guided surgery, photothermal therapy, photodynamic therapy, drug/gene delivery, and combined therapy	cervical cancer therapy	tumor imaging	(330)
32	black quantum dots/mesoporous silica framework/Pt nanoparticles		drug carrier, delivery platform, photodynamic and photothermal therapies	liver cancer therapy	self-compensation mechanisms	(331)
33	polymeric nanoparticles	biocompatible and easy to modify	drug intelligent responses, functional coating	cancer therapy	chemotherapy	(332)
34	cyclodextrin polymer (CDP) based nanoparticles	low toxicity, soluble in water, insoluble in organic solvents, and easy to modify	potential to improve the loading capacity of nanostructured lipid carriers, solid lipid nanoparticles, and liposomes	cancer therapy	chemotherapy	(333)
35	mAb nanoparticles	biocompatible, highly specific; recognize and find specific proteins on cancer cells	used to deliver nanoparticles and improve drug targeting to cancer cells	cancer therapy	chemotherapy	(334)
36	nanoemulsion (NE)	colloidal particulate system in submicrometer size range; these carriers are solid spheres with an amorphous, lipophilic, negatively charged surface	drug delivery, biological or diagnostic agents, safeguard the effective content of drugs from hydrolysis and oxidation	cancer therapy	chemotherapy	(335)

## 6 Current Technological Challenges and Limitations of Effective Theranostics

Smart nanomaterials offer a robust platform for effective cancer theranostics as they can be triggered in response to specific external or endogenous stimuli like pH, temperature, enzymes, or a particular biological molecule. Compared to traditional cancer theranostic approaches, smart nanomaterials based approaches exhibit improved selectivity and sensitivity with fewer adverse effects. Furthermore, an additional benefit of using a theranostic device is the noninvasive, rapid, and precise identification of early chemotherapeutic responses. Overall, using theranostic platforms based on nanotechnology gives chemotherapy unmatched advantages for overcoming its long-accompanied disadvantages.^336^ Early cancer detection, tailored medication delivery, drug discovery, and effective anticancer therapy are all possible with nanotechnology-based techniques and nanomaterials in oncology.^337^ Gold nanoparticles (AuNPs) have been widely used in treating breast and prostate cancers as drug delivery systems. AuNPs are considered because of their dependable qualities, such as their ability to scatter light, absorb light, and convert optical energy into heat.^338^ Though the nanomaterials have proven their anticancer activities. One of the most challenging problems in the field of nanotheranostics is system complexity, having numerous functionalities in a single nanometer particle size. Industrial scale-up production and its clinical translation are also two of the main obstacles in the field of NPs. Scientists and engineers should adopt an interdisciplinary approach to successfully absorb this field in cancer theranostic nanomedicine. With many of their intrinsic molecular properties and multifunctionality, different nanomaterials have recently become an exciting tool in cancer theranostic applications, assisting in successful therapy, diagnosis, and imaging.^339^ However, several obstacles prevent their clinical translation, including systemic toxicity of nanomaterials within the body, nontargeted distribution, complex synthesis, high cost, reduced biocompatibility, stability and reduced photostability.^317,340^ Another major obstacle to the use of nanomaterials in cancer theranostics is the selection of models as most of the research is in and around cell and animal models that may not be the right models to assess the anticancer efficacy of both diagnostic and therapeutic agents, as these models suffer from various degrees of different chemical and physical stresses and might not be representative of those of whole human organs. In the complex human system, it is difficult to replicate a reaction using just one model.

A single model may not mimic the complex human system; however, the possibility to link several of these models which are capable of recapitulating the *in vivo* interaction, extracellular matrix, intercellular signaling, and *in vivo* growth may provide a system that more closely resembles and can harness a better understanding of *in vivo* events. Biomimetic organ/tumor-on-a-chip tools and organoid model systems are possible solutions to imitate *in vivo* conditions of nanocarriers used in cancer patients.^341−343^ First and foremost, developing a low-cost synthesis method and an easy purification procedure is vitally necessary to enable the mass production of nanomaterials. The focus on engineered nanomaterials with high hydrophilicities will open the door to anticancer drug carriers with enhanced tumor therapy efficacy and drug delivery. Last, but not least, the focus should be directed toward delivering genetic material to the cancer cell for effective cancer theranostics. So far, the full potential of nanomaterials in cancer is not fully utilized, as nanotoxicity and bioaccumulation of NPs constrain their broader applicability.

QDs have generated a great deal of attention in various biological domains due to their several significant benefits over conventional dyes in bioimaging. However, these applications face numerous difficulties, including cellular toxicity brought by producing reactive oxygen species and cadmium leakage, which might threaten the patient after the treatment. For example, graphene QDs have been demonstrated to be safe and nontoxic to normal cells. However, extensive *in vivo* toxicity studies are required prior to its administration^344^ in cancer theranostics. Despite QDs’ effectiveness in cancer theranostics, one of the biggest problems is the possibility of nonspecific reticuloendothelial absorption, which lowers the likelihood and effectiveness of these theranostic agents binding to the targeted cancer site.^345^ In this article, we covered the development of many multifunctional platforms for cancer therapy and diagnosis, as well as diverse methods utilized to reduce the toxicity of nanomaterials through the process of hybridization with different biocompatible lipids, proteins, polymers, or nanoparticles. Table 4 summarizes some cancer theranostic agent characteristics, biocompatibility, and translation stages.

**Table 4:** Some Cancer Theranostics Agents and Their Characteristics and Indications

nanoparticles	purpose in cancer treatment	biocompatibility	diagnostic potential	indications (approved and or in clinical phases)	ref
liposome	therapeutics, diagnostics, theranostics	yes	nanocarriers for the delivery of a variety of drugs	under clinical trial/phase II	(346)
lipid NPs	therapeutics, diagnostics, theranostics	yes	negligible toxicity, multifunctional potential, and functionalization flexibility help them to cross different physiological barriers; LNPs can deliver chemotherapeutic medications to tumor tissue by penetrating the vascular endothelial gaps of tumor	phase I clinical trial (NCT00355888) of MBP-426 was completed, with phase II started	(347)
protein NPs	therapeutics, diagnostics, theranostics	yes	natural availability and biocompatibility; physiology makes it amenable to biomedicine and materials science; amphiphilic in nature; albumin has been recognized as a potential carrier for delivering imaging/anticancer medicines to tumor microenvironments	clinical trials completed for albumin and albumin-based NPs currently undergoing clinical studies/phase II	(348)
MOF	therapeutics, diagnostics, theranostics, imaging	yes	high surface areas, adjustable pore size, crystallinity, thermal stability, and easy surface modification; effective cellular uptake, focused molecular targeting, smart nanodrug delivery devices, and drug-responsive behavior	translation to clinical settings; long-term toxicity and biosafety of NMOFs still need to be further evaluated	(349)
carbon-based nanoparticles	therapeutics, diagnostics, theranostics, imaging	yes	carbon-based nanomaterials exhibit several extraordinary properties, such as high surface area, tunable pore structure, and nonreactive and easy surface functionalization, making them suitable for biological applications	FDA has approved over 35 imaging or therapeutic nanoparticles for clinical trials; under preclinical studies and no clinical trials to date	(350)
inorganic nanoparticles (platinum, gold, silica, palladium, silver, iron oxides, zinc oxide, and rare earth oxides)	therapeutics, diagnostics, theranostics, imaging	yes	inorganic NPs have enormous potential as drug carriers, owing to the easy modification of targeting molecules, different stimuli-driven drug release, and effective delivery to target sites; inorganic NPs are investigated in preclinical and clinical studies for the detection, diagnosis, and treatment of many diseases	currently used in clinical practice, gold nanoparticles or nanoshells (NCT00356980, NCT00848042), silica nanoparticles (NCT02106598), and silica–gold nanoparticles (NCT01270139) might hold a greater chance to speed up the translational process	(351)
quantum dots	therapeutics, diagnostics, theranostics, imaging	yes	compact size, high surface area, surface charges, and precision targeting are distinctive features; increases solubility, prolongs the period of retention, and lessens the negative effects of loaded drugs	FDA has approved a clinical trial for Cornell dot or c-dot for melanoma patients	(352)

## 7 Global Opportunities of Smart Nanomaterials in Next Generation Cancer Theranostics

The use of nanomaterials has shown a paradigm shift in cancer theranostics when compared to conventional approaches. Nanotechnology has revolutionized the cancer theranostics field as the sizes of biomolecules such as DNA, hemoglobin, proteins, and enzymes are in accordance with the size of NPs, which leads to enhanced biomedical application owing to their effective interactions. Owing to the unique properties of nanomaterials, a high surface-to-volume ratio lead to a broader surface area for interactions with biological molecules, resulting in increased sensitivity, enhanced selectivity, and shorter reaction times. Quantum dots (QDs), liposomes, micelles, metallic nanoparticles, dendrimers, and polymeric nanoparticles have recently been utilized for cancer therapy and imaging.^353^ Contrarily, the traditional approaches to treating cancer are ineffective and lack selectivity.^354^ As these traditional procedures affect healthy and cancerous cells, opponents of health also dispute their selectivity.^355,356^ The most significant benefit of smart nanomaterials is their ability to overcome these constraints. Nanomedicine advancements have influenced better outcomes for cancer detection and treatment and have opened new avenues for cancer treatment. Nanomaterials have shown improved pharmacokinetics, biocompatibility, tumor targeting, and stability compared to conventional methods. However, numerous uses of nanomaterials are still challenging because of certain restraints such as nonspecific delivery, multistep synthesis, poor bioaffinity, reduced light immovability, and toxicity of nanomaterials inside the living system.^357^ Therefore, enhanced nanomaterial moieties with well-ordered physicochemical and biological characteristics are strongly considered for cancer theranostics.^358^

Recent advancements in smart nanomaterials based cancer theranostic methodologies have been cultivated, and they are exposing recovering sensitivity and selectivity by condensed negative effects in contrast to traditional methods. In cancer treatment, the smart nanomaterials system reacts to the tumor microenvironment (TME) and leftovers in the neutralized form in standard cells, decreasing the side effects and overall toxicities.^359^ Nanomaterials expand drug effectiveness by enhanced sensitivity, light absorbing capacity, drug half-life lengthening, and drug solubility enhancement with long-standing confirming drug discharge.^360,361^ Additionally, smart nanomaterials can also be applied to intensify the healing, drug loading capability, and organized continuous discharge of drugs, and specific and selective biodissemination by engineering their conformation, designing methodologies, morphology, size, and surface chemistry.^362^ Unlike traditional materials, engineered smart nanomaterials can pierce transversely biological obstacles and empower pH, light, and heat based pursuit of malicious cells.^359^ Manufacturing procedures may be fine-tuned to normalize the functionality and specificity of nanomaterials by transforming the chemical configuration, shape (morphology), and size. For cancer-dealing approaches, nanomaterials can overwhelm the stability and solubility of anticancer drugs.^93,363^ Nanomaterials also help in the treatment of persistent tissue by pointing to the cancer spots, delivery of various drugs, and falling drug conflict.^364^ Additionally, to overwhelm the cytotoxic influence of nanomaterials, novel methodologies have been projected to cultivate biocompatible nanomaterials resembling surface reformation with diverse biodegradable fragments. Extensive research studies need to be carried out for engineered hybrid NPs better suited for cancer treatment with specific binding. The size, shape, composition, and surface of the NPs impact the immune system. Though the nanomaterial approaches are more effective than traditional approaches, the clinical efficacy of this therapy is still inadequate, and further research into the safety and tolerance of these novel techniques is required. Exploiting nanomaterial characteristics to improve the target specificity can achieve significant therapeutic efficacy. Though many research studies were carried out for the uses of nanomaterials in cancer theranostics, only a handful of nanomaterials have successfully translated to clinical applications. Extensive research needs to be carried out for the successful implementation of nanomaterials in clinical applications amid tumor heterogeneity, which poses one of the greatest technological challenges to theranostics.

## 8 Conclusions

The quick expansion of theranostics based on nanotechnology has impressively endorsed the uprising of diagnosis methodologies and cancer oncotherapy. The present review discusses the most relevant advancements in smart nanomaterials, from organic, inorganic, and carbon-based nanoparticles for cancer theranostic applications. The review article’s findings are incredibly inspiring and strongly urge the exploration of nanomaterials as nanocarriers, and their physicochemical attributes can be harnessed to improve *in vivo* performances in cancer theranostics. This review also detailed the limitation and global opportunities for the successful implementation of nanomaterials-based cancer theranostics, which can be translated into clinical applications. One can expect that, by giving attention to the key challenges mentioned above, the nanomaterials-based cancer theranostic platform will bring many exciting opportunities for therapy and diagnosis to achieve accurate cancer diagnoses and address the challenges of conventional oncotherapy. Nanotechnology has demonstrated a new dimension for cancer theranostics by delivering tiny molecules for cancer detection, diagnosis, and therapy. Many different types of cancer are treated using cancer medicines that are based on the exceptional qualities of NPs. With advancement in this vital field, nanomaterials in their different forms have proved to possess enhanced drug delivery effectiveness for cancer. Perfect nanotheranostic arrangements are projected to be (1) harmless and biologically tuned, (2) greatly unwavering and effectual for drug stuffing, (3) simple to modify and prepare, and (4) targeting malignant cells and active for endocytosis.

Furthermore, the groundwork and superiority mechanism of nanoparticles are still complex. Last, the equilibrium between the reliability and efficiency of nanotheranostic settings would be reflected, though the overview of numerous agents in one theranostic stage may fetch various purposes. It is anticipated that, with the advancement in proteomics research on the mechanism of cancer origin, cancer heterogeneity, and multidrug resistance behavior, a large number of NP-based drugs can be exploited. Though extensive research was carried out on NPs, only a few NP-based medications are presently in use, a smaller number are in clinical trials, and the majority are still in the infancy stage. An interdisciplinary approach is required to realize nanomaterials in cancer therapy. Though the application of nanomaterials is in the midst of development, this can be proved to be a new and promising platform that can bring new hope not only for the diagnosis of cancer but also for imaging, treating, and preventing cancer owing to their small sizes, their functionalization potentiality, and the ability to introduce multiple therapeutic agents on their surfaces. This will be a paradigm shift in the way that we treat cancer. Nevertheless, the nanosystems could be too intricate for bulk-scale engineering. Since present research and development efforts have placed a cumulative effect on these nanosystems, innovation in materials science integrated with biological material may accomplish more real-world presentations of nanotheranostic platforms in cancer diagnostics.

In conclusion, the rapid advancement in theranostics based on nanotechnology has significantly acelerated the revolution in cancer oncotherapy and diagnosis. The best nanotheranostic systems should be (1) nontoxic and biocompatible, (2) very stable and effective in drug loading, (3) easy to prepare and modify, and (4) capable of tumor targeting and endocytosis. Although the majority of the nanoparticles listed above do not appear to have obvious cytotoxicity, additional experiments are required to ensure safety in clinical translations. Moreover, nanoparticle preparation and quality assurance are still challenging tasks. Notably, the synthesis of organic nanomaterials is frequently laborious with low yields. Last, but not least, although combining several agents in a single theranostic platform may bring about a variety of activities, it is important to balance the efficiency and dependability of nanotheranostic systems. However, the nanosystem could be too complicated for large-scale synthesis. We presuppose that the revolution in clinical translation for NP-based cancer therapy with rational approaches dealing with synthetic strategies, toxicity, and cellular and physiological factors will be accomplished with nanotechnology and cancer therapy development. Laboratories are coming up with nanomedicine-based drug delivery systems with a strong emphasis on cutting-edge technological and scientific advancements that succeed on a small scale. These laboratories often are familiar with the technical problems that occur in the industry for the commercializing processes. A strong collaboration among pharmaceutical companies and academic laboratory groups must be established to bridge this gap. Nevertheless, current investigators are emphasizing these difficulties, and several initiatives are being taken to achieve more practical applications of nanotheranostic platforms in the treatment of cancer.

## Abbreviations

PAT: photoacoustic therapy
PDT: photodynamic therapy
PEI: poly(ether imide)
PEG: polyethylene glycol
PET: positron emission tomography
CT: computed tomography
MRI: magnetic resonance imaging
P-gp: P-glycoprotein
PL: peptide lipid
PTEN: phosphatase and tensin homologue
PTT: photothermal therapy
PTX: paclitaxel
ROS: reactive oxygen species
SWNTs: single-walled carbon nanotubes
TME: tumor microenvironment
TNF:α: tumor necrosis factor-α
TPGS: d-α-tocopheryl polyethylene glycol succinate
TPP: triphenylphosphine.
EMT: epithelial–mesenchymal transition
DOX: doxorubicin
CDs: carbon dots
IRMOF: isoreticular metal–organic framework
ZIF-8: zeolitic imidazolate framework-8
UiO: Zr-based metal–organic framework
HfDBP: hafnium/5,15-di(*p*-benzoato)porphyrin
ZIF: zeolitic imidazolate framework
NMOF: nanovesicular metal–organic framework
TBP: 5,10,15,20-tetra(*p*-benzoato)porphyrin
ZnP: zinc phthalocyanine
TBB: 5,10,15,20-tetra(*p*-benzoato)bacteriochlorin
QC: 2″,3′-dinitro-[1,1′:4′,1″;4″,1‴-quaterphenyl]-4,4‴-dicarboxylate
MOF-199: Cu(II) carboxylate based metal–organic framework
MTT: 3-(4,5-dimethylthiazol-2-yl)-2,5-diphenyl-2*H*-tetrazolium bromide
